# Hyaluronan control of the primary vascular barrier during early mouse pregnancy is mediated by uterine NK cells

**DOI:** 10.1172/jci.insight.135775

**Published:** 2020-11-19

**Authors:** Ron Hadas, Eran Gershon, Aviad Cohen, Ofir Atrakchi, Shlomi Lazar, Ofra Golani, Bareket Dassa, Michal Elbaz, Gadi Cohen, Raya Eilam, Nava Dekel, Michal Neeman

**Affiliations:** 1 Department of Biological Regulation, Weizmann Institute, Rehovot, Israel.; 2 Agricultural Research Organization, Volcani Center, Israel.; 3 Department of Gynecology, Tel Aviv Sourasky Medical Center, Affiliated to the Sackler School of Medicine, Tel Aviv University, Israel.; 4 Department of Pharmacology, The Israel Institute for Biological Research, Nes Ziona, Israel.; 5 Department of Life Sciences Core Facilities and; 6 Department of Veterinary Resources, Weizmann Institute, Rehovot, Israel.

**Keywords:** Angiogenesis, Reproductive Biology, Embryonic development, Mouse models

## Abstract

Successful implantation is associated with a unique spatial pattern of vascular remodeling, characterized by profound peripheral neovascularization surrounding a periembryo avascular niche. We hypothesized that hyaluronan controls the formation of this distinctive vascular pattern encompassing the embryo. This hypothesis was evaluated by genetic modification of hyaluronan metabolism, specifically targeted to embryonic trophoblast cells. The outcome of altered hyaluronan deposition on uterine vascular remodeling and postimplantation development were analyzed by MRI, detailed histological examinations, and RNA sequencing of uterine NK cells. Our experiments revealed that disruption of hyaluronan synthesis, as well as its increased cleavage at the embryonic niche, impaired implantation by induction of decidual vascular permeability, defective vascular sinus folds formation, breach of the maternal-embryo barrier, elevated MMP-9 expression, and interrupted uterine NK cell recruitment and function. Conversely, enhanced deposition of hyaluronan resulted in the expansion of the maternal-embryo barrier and increased diffusion distance, leading to compromised implantation. The deposition of hyaluronan at the embryonic niche is regulated by progesterone-progesterone receptor signaling. These results demonstrate a pivotal role for hyaluronan in successful pregnancy by fine-tuning the periembryo avascular niche and maternal vascular morphogenesis.

## Abstract 2

Hyaluronan fine-tunes the periembryo avascular niche and maternal vascular morphogenesis during implantation.

## Graphical abstract

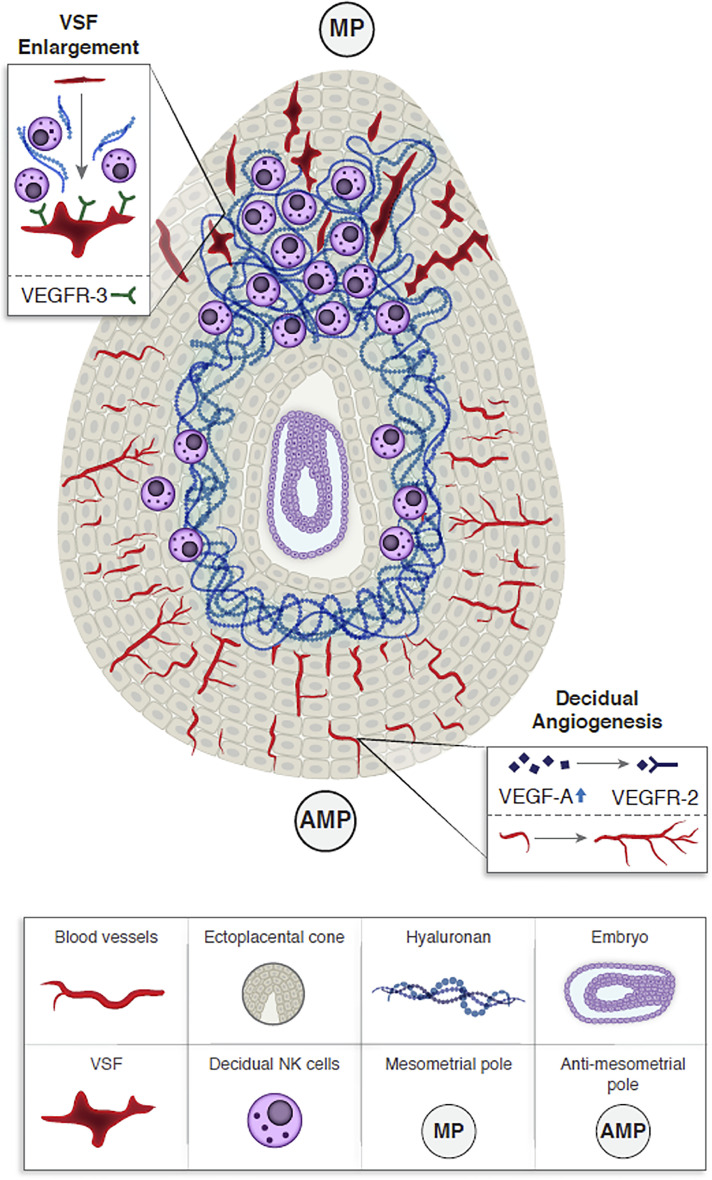

## Introduction

The birth of a properly developed mammalian offspring requires the fulfillment of a series of complex, highly regulated processes, initiated by embryo implantation. In humans, the success of natural conception per menstrual cycle is low (~30%), and implantation defects were implicated in 75% of failed pregnancies (1, 2). Implantation occurs at the blastocyst stage, at which the first cell lineages form (3). The blastocyst contains an inner cell mass (ICM), which mainly gives rise to the fetal organs, and an outer epithelial-like cell layer, the trophectoderm, which will form the extra embryonic tissues including the placenta. Implantation in mice, which takes place at E4.5, is preceded by blastocyst apposition and its attachment to the uterine epithelium, to trigger decidualization of the stromal cells at E4.0 (4), characterized by rapid cellular proliferation and differentiation. Secretion of VEGF-A by the decidual cells and induction of VEGF/VEGFR-2 signaling (5, 6), result in an immediate local increase in vascular permeability followed by the expression of CD34, an endothelial marker for angiogenesis (7). This neovascularization event takes place in the antimesometrial pole of the decidua, the site of initial trophoblast invasion, and is governed by the progesterone-progesterone receptor (PR) axis (5). The newly formed vessels continue to increase in number and diameter (8). The decidual mesometrial pole, on the other hand, is characterized by the development of vascular sinuous folds (VSFs), which are arterio-venous vascular shunts, markedly enlarged and elongated before placentation (5). Another receptor for VEGF signaling, VEGFR-3, participates in this orchestrated series of events by inhibiting VEGFR-2 expression, consequently blocking VEGF/VEGFR-2–induced permeability (9, 10). Perturbation of maternal vascular remodeling during early pregnancy is associated with pathologies, often detected at later stages of gestation, including first-trimester miscarriages, preeclampsia, placental failure and intrauterine growth restriction (5).

Effective delivery of maternal supply to the embryo is further dependent on the formation of 2 decidual subcompartments at E6.5; the avascular primary decidual zone, adjacent to the embryo, and the highly vascularized secondary decidual zone at the decidual rim. This spatially regulated growth of vessels, together with the embryonic diffusion barrier, form the hypoxic niche of the periimplantation embryo (11). This barrier enables nourishing of the embryo while avoiding its immediate exposure to the maternal circulation, thus controlling the delivery of blood-borne high–molecular weight contents, including immune cells and immunoglobulins (12, 13). The mechanism underlying the formation of this diffusion barrier is poorly understood.

Prior to placentation, the mesometrial decidua, is enriched with innate immune cells, composed mainly of uterine NK cells, accounting for approximately 30% of all decidual leukocytes (14, 15). Interestingly, in both humans as well as mice, the functions of uterine NK cells differ profoundly from peripheral NK cells. Namely, unlike the predominant cytotoxic actions against virus-infected or cancerous cells, characteristic to peripheral NK cells, the uterine NK subset shows differential effector repertoire and vessel remodeling activities (15–17). Moreover, uterine NK cells have been demonstrated to regulate “ligand-independent” VEGFR-3 coordination of enlargement and elongation of VSFs via pruning of mesometrial vasculature (5).

Implantation is also characterized by modulations of extracellular matrix (ECM) components, including specific mucins, selectins, integrins, and glycosaminoglycans, such as heparan sulfate and hyaluronan (1). Trophoblast invasion to the uterine stroma initiates the establishment of direct contacts between the trophoblast cells and the uterine decidual cells, as well as decidual blood vessels and their supporting ECM (18). The ECM undergoes extensive transformations, including generation of an interrupted and thickened basement membrane within the pericellular spaces of the postimplantation decidua, which confines infiltration of immune cells and blocks exposure to maternal antibodies within the frame of primary maternal tolerance (19).

Hyaluronan is a negatively charged unbranched cell surface–associated polysaccharide, composed solely of repeating disaccharides units of [D-glucuronic acid-β1,3-N-acetyl- D-glucosamine-β1,4-]_n_ (20). Hyaluronan synthesis is catalyzed by 3 hyaluronan synthases, HAS-1, HAS-2, and HAS-3, all anchored to the plasma membrane. The produced hyaluronan is extruded in its intact polysaccharide form in a typical molecular mass of 1 × 10^6^ to 1 × 10^7^ Da (21). Hyaluronan, in its high–molecular weight form, inhibits angiogenesis in a size-dependent manner (22–25). In the female reproductive tract, hyaluronan is a major component of cumulus cell mucification before ovulation, as well as a regulator of follicular vascular remodeling at ovulation; it also takes part in uterine decidualization (22, 26, 27).

Hyaluronan is cleaved by hyaluronidases. In both humans and mice, there are 6 hyaluronidase genes encoding for enzymes with different enzymatic properties and cellular localizations (20, 28). The main enzymes studied are Hyal-1 and Hyal-2. The most abundant plasma and urine enzyme, Hyal-1, intracellularly degrades hyaluronan to small oligosaccharides (21). Hyal-2, which is anchored to the plasma membrane by a glycosylphosphatidylinositol link, hydrolyzes only high–molecular weight hyaluronan (21), and generates intermediate hyaluronan fragments of approximately 20 kDa. These fragments are characterized by their proangiogenic activity, including induction of endothelial cell proliferation and tube formation, by mechanisms involving VEGF release and upregulation of VEGFR-2 expression (23, 25, 28–31). Hyaluronan oligosaccharides are also potent inducers of MMP-9 activity (32, 33) and stimulators of proinflammatory macrophages (21, 34–36). MMP-9 can independently induce angiogenesis by releasing the ECM-bound VEGF, potentially increasing its abundance near VEGFR-2–expressing cells (37).

In the study presented here, the role of hyaluronan as a vascular morphogen shaping the primary maternal-embryo barrier was evaluated during implantation and early mouse pregnancy. To this end, we employed lentivirus-mediated genetic modification of hyaluronan metabolism, directed at the embryonic trophectoderm. We report herein that disruption of hyaluronan synthesis, as well as its increased cleavage at the embryonic niche, impairs implantation by induction of decidual vascular permeability, defective VSF formation, and breach of the maternal-embryo barrier. This phenotype was associated with elevated MMP-9 expression and disturbed uterine NK cell recruitment and function. Conversely, enhanced deposition of hyaluronan resulted in the expansion of the maternal-embryo barrier and increased diffusion distance, leading to compromised implantation. We also demonstrate that deposition of hyaluronan at the embryonic niche is subjected to regulation of the PR. These results demonstrate a pivotal role for hyaluronan in the success of pregnancy by fine-tuning the periembryo vascular morphogenesis, so as to maintain the primary maternal-embryo vascular barrier and the hypoxic avascular niche during early pregnancy.

## Results

### Decidual angiogenesis spatially and temporally mirrors the pattern of deposition of hyaluronan and its degradation products.

**Figure 1:**
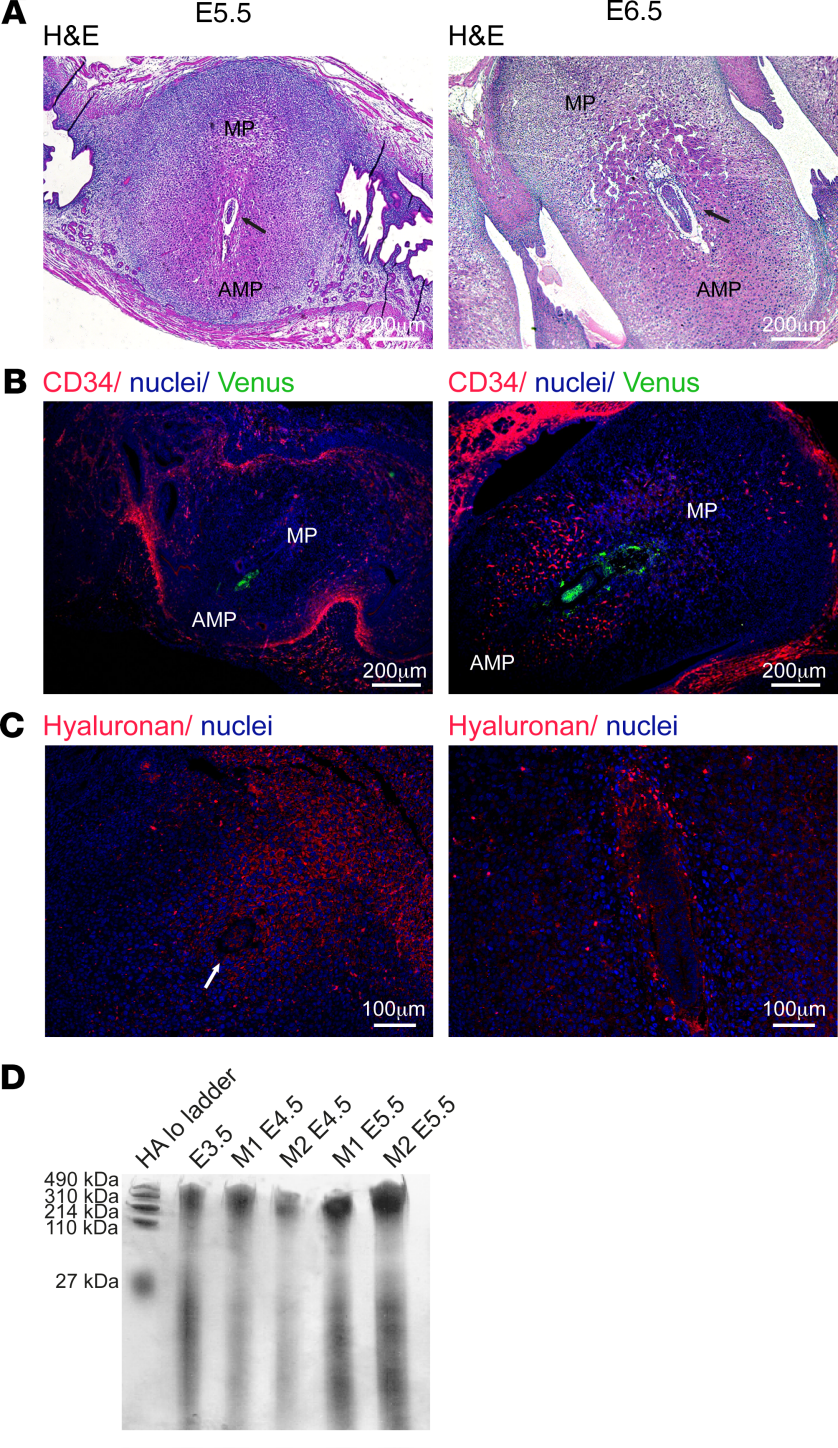
Hyaluronan deposition and vascular remodeling in the implantation site during early pregnancy. Female mice were mated with Venus^+^ males, and their uterine horns were harvested after implantation (E5.5–E6.5, *n* = 4 dams). (**A**) H&E staining of decidua. Black arrows designate embryos. (**B**) Newly formed CD34^+^ uterine blood vessels, reflecting decidual angiogenesis. (**C**) Hyaluronan localization indicated by IHC. White arrow designate embryo. (**D**) Glycosaminoglycans were separated from uteri of pregnant mice, at different time points, subjected to native PAGE next to hyaluronan standard and stained for hyaluronan (*n* = 2 dams; 3 implantation sites per dam). AMP, antimesometrial pole; MP, mesometrial pole.

Following embryo implantation, the uterus undergoes rapid and profound tissue remodeling (Figure 1A). The previously reported angiogenic process at this stage (7) is herein indicated by CD34, a marker for newly formed blood vessels observed in the antimesometrial pole of the decidua (Figure 1B). Interestingly, this vascular modification coincided spatially and temporally with dynamic alterations in the deposition of hyaluronan. Specifically, following implantation (E5.5), hyaluronan accumulated in the mesometrial pole surrounding the embryo. At E6.5, hyaluronan was mainly deposited in the ectoplacental cone in mesometrial orientation to the implantation chamber (Figure 1C). Overall, the accumulation of hyaluronan oligosaccharides was detected in pregnant mice following embryo implantation, in concomitance with intensified decidual angiogenesis (Figure 1D).

To further explore hyaluronan deposition, we analyzed the distribution of hyaluronan synthases, hyaluronidases, and hyaluronan binding proteins throughout the periimplantation period. We found that the hyaluronan synthesizing enzyme HAS-1 was locally upregulated in implantation sites at E4.5, transiently decreasing at E5.5, and increasing again at E6.5 (Supplemental Figure 1A; supplemental material available online with this article; https://doi.org/10.1172/jci.insight.135775DS1). Interestingly, the expression of HAS-2 continuously decreased during the postimplantation period (Supplemental Figure 1B). Alongside the trend of hyaluronan accumulation, the expression of the degrading Hyal-1 underwent gradual downregulation (Supplemental Figure 1C), whereas the levels of Hyal-2 remained stable throughout implantation (Supplemental Figure 1D). We also analyzed the expression pattern of 2 hyalhedrines, hyaluronan ECM stabilizing glycoproteins TSG-6 and Versican. TSG-6 was significantly upregulated on the day of implantation, followed by a later, sharp reduction in its expression (Supplemental Figure 1E), whereas Versican expression levels remained constant during the 2 consecutive days following blastocyst attachment and invasion (Supplemental Figure 1F).

**Figure 2:**
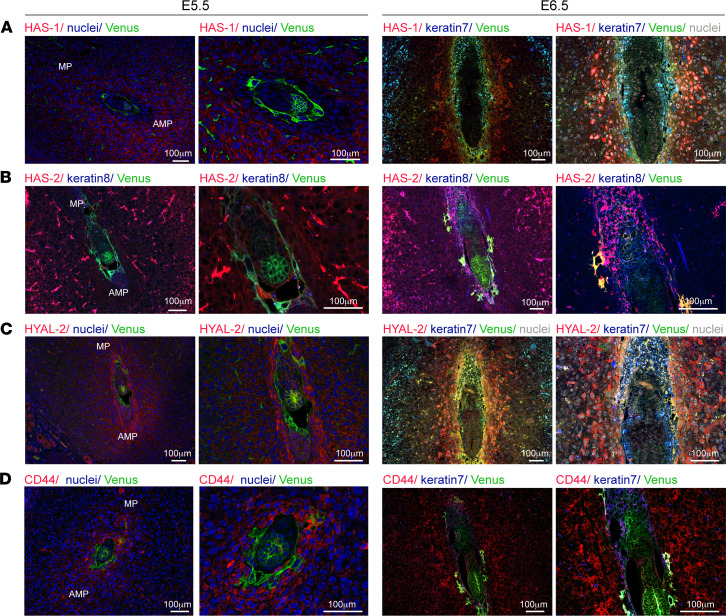
Hyaluronan metabolism following embryo implantation. Female mice were mated with Venus^+^ males, and their uterine horns were harvested after implantation (E5.5–E6.5) and subjected to histological analysis. Venus (embryo) and keratin detects ectoplacental trophoblasts and visceral endoderm cells (*n* = 4 dams, 10 implantation sites). Right panels are magnifications of left panels. (**A** and **B**) IHC analysis of hyaluronan synthesizing enzymes HAS-1 and HAS-2. (**C**) IHC analysis of Hyal-2, hyaluronan degrading enzyme. (**D**) IHC analysis of the most prominent hyaluronan receptor, CD44.

For further resolution, we examined the spatiotemporal distribution of hyaluronan biosynthesis enzymes in the different decidual subcompartments (Figure 2). This analysis revealed that, at E5.5 after implantation, HAS-1 was expressed solely by maternal cells in the primary decidual zone, forming a sphere surrounding the embryo, while HAS-2 was expressed both by the trophoblast giant and the cytotrophoblast cells at the ectoplacental cone. At E6.5, both hyaluronan synthases were distributed in the antimesometrial pole of the decidua. HAS-1 was expressed in maternal cells adjacent to trophoblast cells, while HAS-2 was detected robustly in the ectoplacental cone at the mesometrial region of the embryonic egg cylinder and by the trophoblast giant cells (Figure 2, A and B).

At E5.5, hyaluronan degrading enzymes Hyal-2 and Hyal-1 were both detected in the maternal primary decidual zone, while Hyal-2 was also expressed by all subtypes of trophoblast cells. At E6.5, hyaluronidases were expressed mainly by the trophoblast giant cells and the adjacent decidualized cells, with a lesser extent by extraembryonic endoderm cells (Figure 2C and Supplemental Figure 1G).

Not surprisingly, 2 of the receptors for hyaluronan oligosaccharides, CD44 and LYVE-1 (20), were coexpressed with HAS-2 and the 2 hyaluronidases. After the day of implantation, both CD44 and LYVE-1 showed a pattern of expression similar to that of Hyal-2 and Hyal-1 (Figure 2D and Supplemental Figure 1H). Interestingly, RHAMM, a hyaluronan-mediated motility receptor, was highly expressed by the maternal decidualized cells in a ring-like structure at E5.5, as well as in primary decidualizaed cells adjacent to the ectoplacental cone at E6.5 (Supplemental Figure 1I).

Overall, we show the close spatial association of hyaluronan enzymatic biosynthesis — and degradation, as well as the receptors for its degradation products — at the feto-maternal interface after implantation.

### PR signaling positively regulated hyaluronan degradation, trophoblast invasion, and decidual angiogenesis.

**Figure 3:**
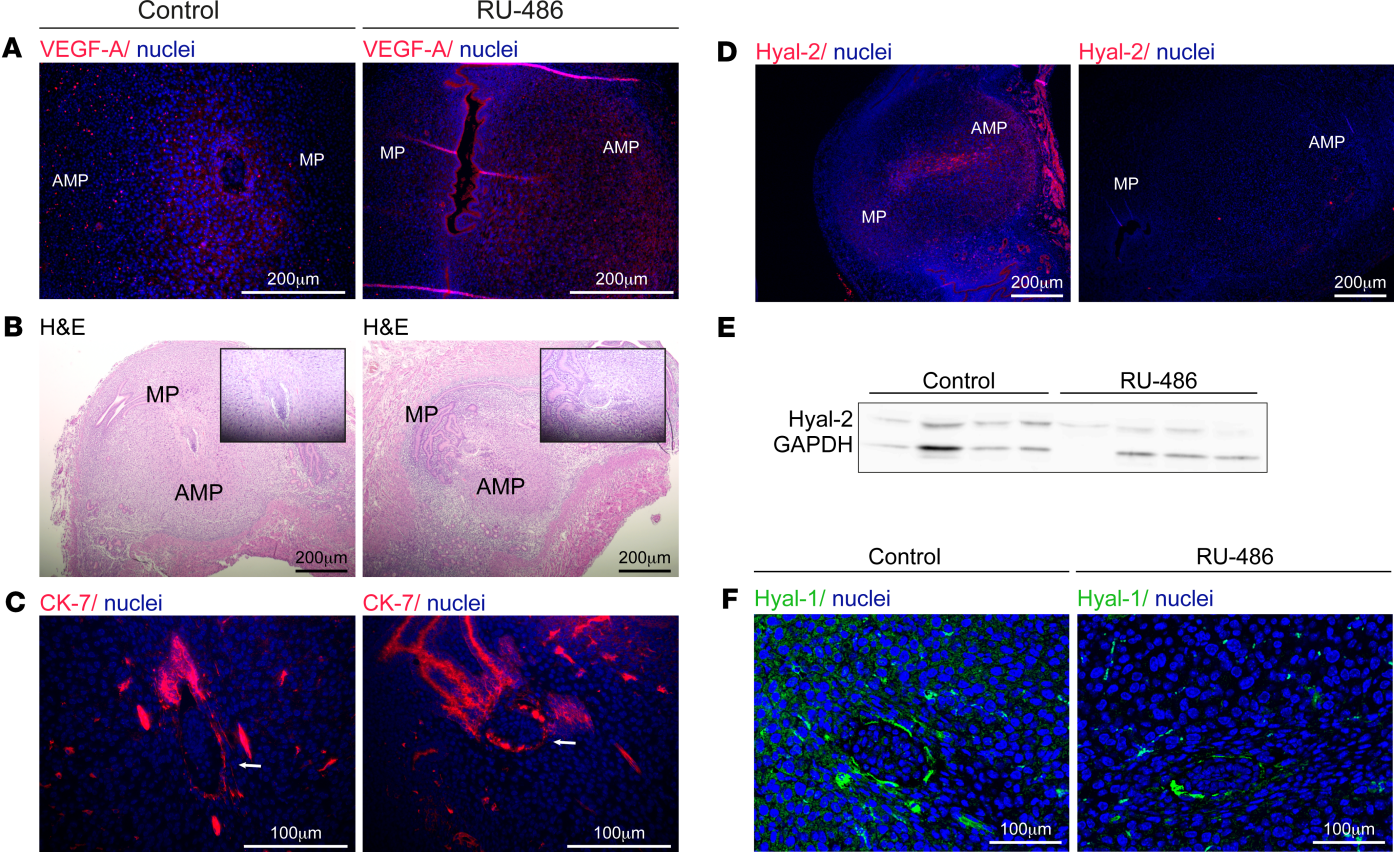
Pharmacological blockade of the progesterone receptor after attachment. Female ICR mice were mated with ICR males and administered with RU-486 at E4.5. Their uterine horns were harvested at E5.5 (*n* = 3 dams). (**A**) Representative images of VEGF-A staining in E5.5 decidua. (**B**) Smaller decidua and abnormal embryonic morphology, detected by H&E staining. (**C**) Comparison of cytokeratin-7–expressing trophoblast cells and their distribution; white arrows indicate embryos. (**D**) Representative images of decreased Hyal-2 decidual distribution. (**E**) Western blot analysis of Hyal-2 following RU-486 treatment (*n* = 4 dams; pools of 3 decidua). (**F**) Representative images of Hyal-1 staining in E5.5 decidua.

To examine the involvement of progesterone in hyaluronan metabolism, we suppressed PR signaling via a single administration of its antagonist RU486 at E4.5 after implantation (8 mg/kg body weight). As previously demonstrated for later pregnancy (5), administration of RU486 at E5.5, soon after initial decidualization, resulted in a decreased VEGF-A expression (Figure 3A). Downregulation of VEGF-A was accompanied in our study by resorptions of implanted embryos (Figure 3B) and impaired antimesometrial stromal invasion by primary trophoblast giant cells (Figure 3C). Confirming a previous report of progesterone-regulated hyaluronan degradation (38), we found that RU486 treatment led to decreased expression of the 2 hyaluronidases Hyal-2 (Figure 3, D and E) and Hyal-1 (Figure 3F).

### Hyal-2 overexpression in trophoblast cells disrupted the hyaluronan maternal-embryo barrier, leading to embryo resorption.

**Figure 4:**
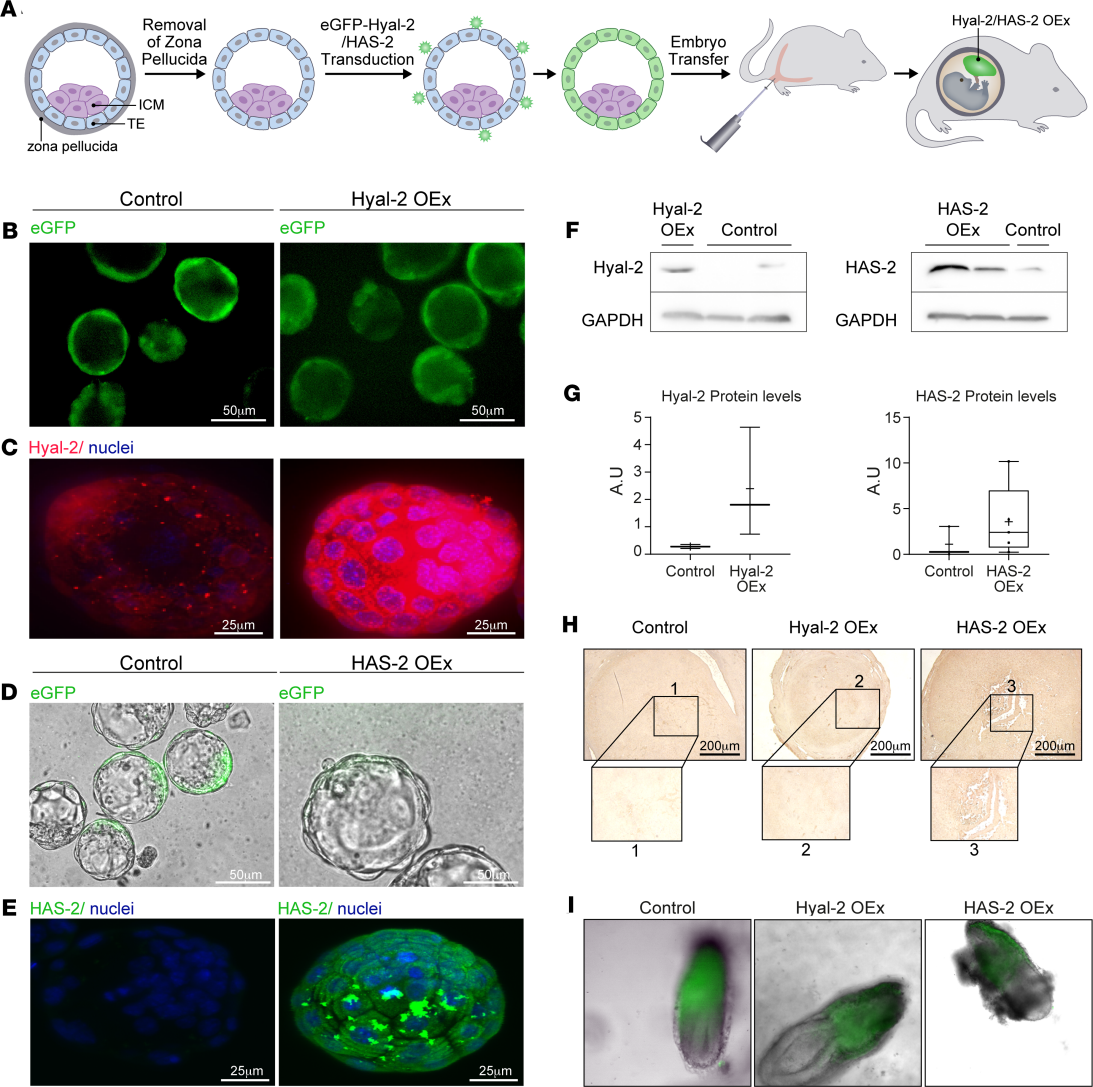
Genetic modifications in embryonic trophoblast cells. (**A**) Morulae were retrieved from pregnant mice (E2.5), grown to blastocysts, infected with lentiviral vectors, and transferred to pseudopregnant mice. Immediately thereafter, Hyal-2 was overexpressed in the trophectoderm. (**B**) eGFP expression prior to embryo transfer. (**C**–**E**) Maximal intensity projections of whole-mount immunofluorescence for Hyal-2 in blastocysts following lentiviral transduction prior to embryo transfer, blastocysts over-expressing HAS-2 in their trophectoderm visualized for eGFP expression, and HAS-2 overexpression following viral infection. (**F**) Validation of Hyal-2 and HAS-2 overexpression by Western blot analysis. (**G**) Quantification of Western blot analysis following infection with lentiviral vectors. (**H**) Histological assessment of hyaluronan deposition as a result of Hyal-2 and HAS-2 overexpression. (*n* = 3 mice). (**I**) E6.5 trophoblast cells exclusively expressing eGFP alongside the WT epiblast and endoderm.

To study the role of hyaluronan at the feto-maternal interface during the postimplantation period, we used lentiviral infection to generate blastocysts expressing EGFP in their trophectoderm cells, along with overexpression of Hyal-2 (Figure 4A). Prior to their transfer to pseudopregnant recipient mice, the blastocysts were visualized for EGFP expression. Notably, fluorescence was restricted to the trophectoderm and could not be detected in the ICM (Figure 4, B and D). Transgene overexpression was validated by immunofluorescence of infected embryos (Figure 4, C and E), as well as by Western blot analysis (Figure 4, F and G). Hyaluronan was modified as expected following implantation in foster dams (Figure 4H). Lentiviral transduction of trophoblasts visualized by EGFP remained effective following implantation (Figure 4I), without notable interference with embryonic development, as previously demonstrated (39).

**Figure 5:**
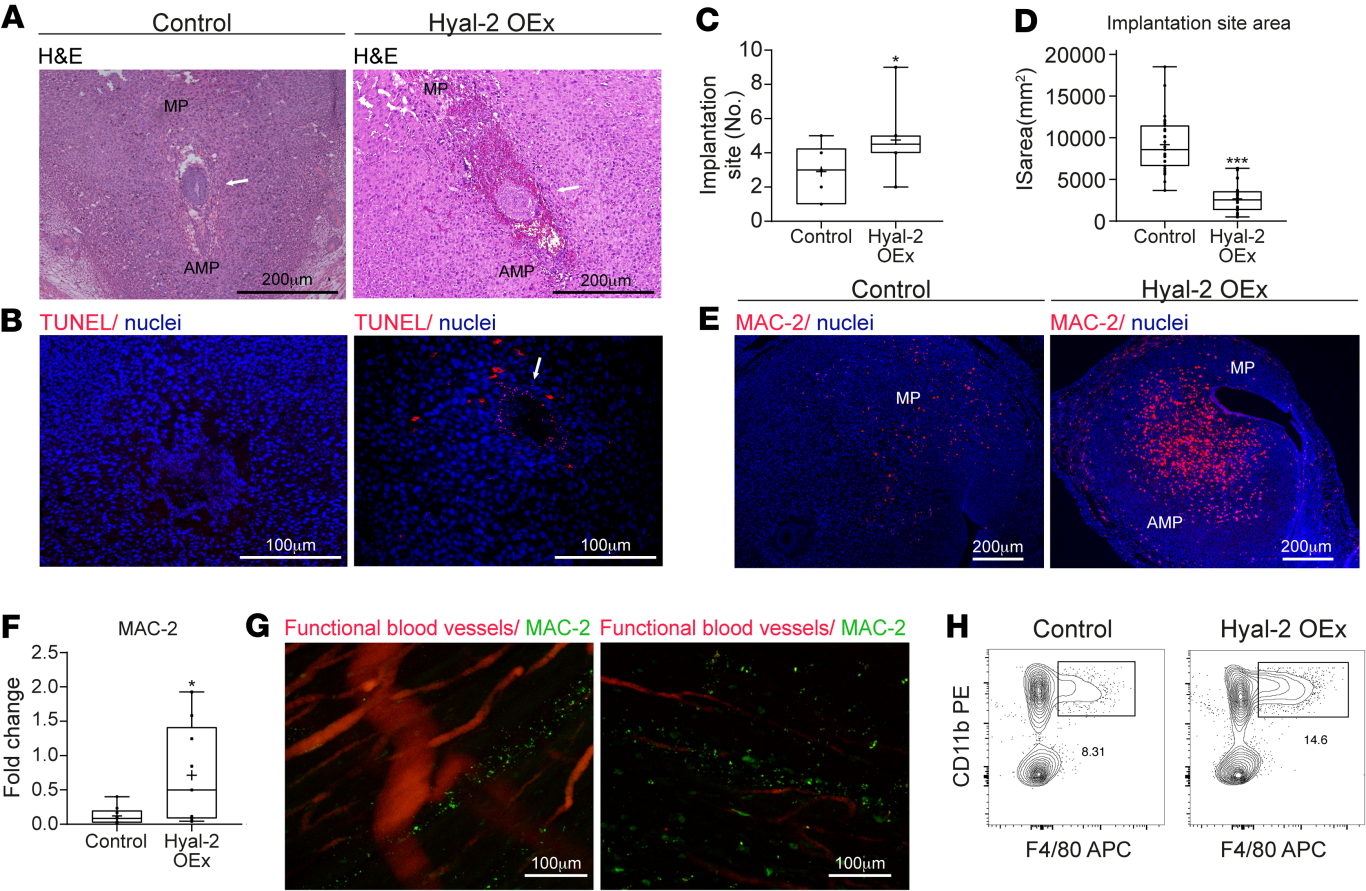
Trophectoderm overexpression of Hyal-2 resulted in enhanced implantation rate but early embryonic lethality. Implantation sites were studied at E6.5, containing sham-infected embryos and embryos overexpressing Hyal-2 in their trophoblast. (**A**) H&E staining (*n* = 6 dams; white arrows indicate decidua). (**B**) TUNEL staining revealed cell death at the embryonic niche of Hyal-2 OEx (white arrow indicate embryonic cells; *n* = 4 dams). (**C** and **D**) Quantification of observed decidua, as well as their calculated area (2.9 ± 0.5; 4.75 ± 0.7; *P* = 0.02) (*n* = 12 dams in control, *n* = 10 dams in Hyal-2 OEx) divided by (3.42-fold change± 0.07; 0.03; *P* = 0.0006) (*n* = 8 dams, 27 implantation sites in control; *n* = 6 dams, 23 implantation sites for Hyal-2 OEx). (**E** and **F**) Detection of MAC-2^+^ macrophages and their quantification (5.9-fold change ± 0.35; *P* = 0.02) (*n* = 5 dams, 9 decidua in each group). (**G**) Presence of MAC-2^+^ macrophages was further demonstrated using immunostaining of macrophages in implantation sites harvested from surrogate mothers injected with rhodamine-labeled (ROX-labeled) lectin. Tissues were made transparent using modified tissue clearing procedure, thus enabling visualization of whole decidua by confocal microscopy. (*n* = 2 dams from each group). (**H**) Flow cytometry analysis of CD11b^+^F4/80^+^ cells in E6.5 decidua (12.33 ± 0.31 [control]; 17.02 ± 1.49 [overexpression]; *P* = 0.04) (*n* = 3 dams in control, *n* = 5 dams in Hyal-2 OEx). The statistical analysis applied was Student’s *t* test (**C**, **D**, **F**, and **H**).

Overexpression of Hyal-2 in trophoblast cells resulted in augmented mesometrial accumulation of maternal blood (Figure 5A). Multiple embryo resorptions and embryonic cell death were observed in pregnant mice carrying Hyal-2–overexpressing (Hyal-2 OEx) blastocysts (Figure 5B and Supplemental Figure 2G). Interestingly, individual decidua were reduced in diameter, whereas their total number in these mice was significantly higher, compared with controls (Figure 5, C and D). This manipulation was accompanied by increased MMP-9 levels in the blastocysts before implantation (Supplemental Figure 2, A and B). After implantation, development of Hyal-2 OEx embryos resulted in robust activation of macrophages, demonstrated by staining for MAC-2 — a marker for inflammatory macrophages — visualized by IHC and by tissue clearing (Figure 5, E–G), as well as increased accumulation of F4/80 macrophages, which was detected by FACS analysis (Figure 5H).

**Figure 6:**
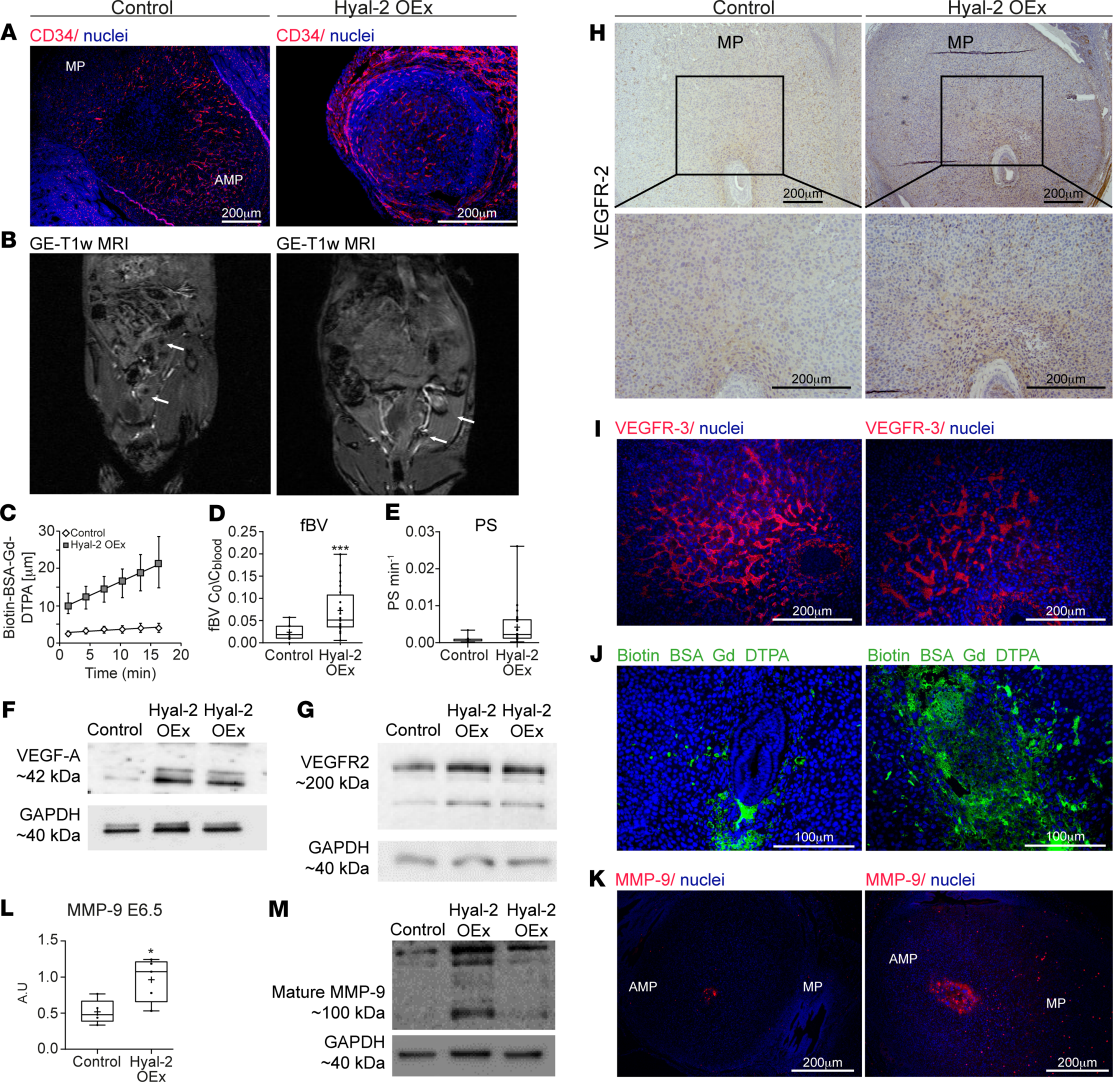
Detrimental decidual hypervascularity and breach of the maternal-embryo barrier is induced by trophectoderm Hyal-2 overexpression. (**A**) Ectopic presence of newly formed maternal blood vessels in the embryonic niche was observed in dams carrying Hyal-2 OEx embryos (*n* = 5 dams). (**B**) GE-T1–weighted MRI images of decidua, acquired from pregnant mice at E6.5, 3 minutes after administration of biotin-BSA-GdDTPA; white arrows point at implantation sites. (**C–E**) Linear regression plots from DCE MRI analysis. Decidual blood volume fraction (fBV; 0.023 ± 0.01 [control]; 0.072 ± 0.009 [overexpression], *P* = 0.0005) and permeability (PS; 0.00092 ± 0.0003; 0.0074 ± 0.0031, *P* = 0.09) were calculated from DCE MRI (*n* = 5 dams 13 implantation sites in control; 6 dams 24 implantation sites in Hyal-2 OEx). (**F** and **G**) Increased levels of VEGF-A and VEGFR-2 in decidua harvested at E6.5 (*n* = 3 dams, 2 implantation sites from each group). (**H**) Increased VEGFR-2 expression on decidual vessels in mesometrial orientation, as well as in cytotrophoblast cells, in Hyal-2 OEx foster dams (*n* = 4 dams in each group). (**I**) Declined VEGFR-3^+^ VSFs endothelial expression, demonstrated by immunofluorescence (*n* = 3 dams, 6 implantation sites from each group). (**J**) Hyperpermeable blood vessels in the embryonic niche were visualized by staining of biotin-BSA-GdDTPA, 40 minutes after intravenous injection (*n* = 5 dams). (**K**) Increased MMP-9 levels in E6.5 trophoblast cells OEx Hyal-2 (*n* = 5 dams in each group). (**L**) Quantification of MMP-9 expression in E6.5 decidua by immunofluorescence of histological sections (1.858-fold change ± 0.14 [control]; 0.25 [overexpression]; *P* = 0.01) (*n* = 5 dams in each group). (**M**) Increased levels of pro MMP-9 and mature MMP-9 in decidua harvested at E6.5 (*n* = 3 dams, 2 implantation sites in each group). The statistical analysis applied was Student’s *t* test (**D**, **E**, and **L**).

Interestingly, Hyal-2 OEx resulted in augmented angiogenesis in the embryonic niche (Figure 6A). MRI inspection of pregnant mice carrying Hyal-2 OEx blastocysts detected enhanced accumulation of i.v. injected biotin-BSA-GdDTPA as early as 3 minutes after its administration (Figure 6B). Further analysis revealed a significant increase in blood volume fraction, with no change in vessel permeability (Figure 6, C–E). The Hyal-2 OEx embryos expressed an increased levels of VEGF-A and an upregulated VEGFR-2, the latter observed specifically in mesometrial orientation to the embryo and in the ectoplacental cone (Figure 6, F–H). Notably, VEGFR-1 levels did not change following hyaluronan-increased degradation (Supplemental Figure 2C). However, Hyal-2 OEx in the trophoblast brought about a decreased VEGFR-3 expression in the mesometrial pole. This last effect resulted in diminished VSF formation (Figure 6I and Supplemental Figure 2D), accompanied by periembryo mesometrial contrast agent accumulation (Figure 6J). Thus, enhanced enzymatic degradation of hyaluronan was sufficient for breaching the primary maternal-embryo vascular barrier and perturbing the spatial balance of decidual-vascular remodeling. Overexpression of Hyal-2 resulted in increased MMP-9 expression in postimplantation trophoblast cells (E6.5). The expression of MMP-9 was examined by immunofluorescence, as well as by Western blot analysis (Figure 6, K–M). As expected, this increased expression was accompanied by excess invasion of trophoblast cells at E6.5 (Supplemental Figure 2, E and F).

### Clearance of hyaluronan prominently modified the decidual immune milieu.

Overexpression of Hyal-2 in the trophoblasts led to increased CD45^+^ immune cells in their respective implantation sites. Nevertheless, the observed increase in CD11b^+^F4/80^+^ macrophages (Figure 5H) and other populations of mononuclear phagocytes harboring the decidual mesometrial pole (i.e., CD11b^+^CD11c^+^ DCs and CD11b^+^Ly6c^+^ monocytes) were not affected by hyaluronidase overexpression in the feto-maternal interface (Supplemental Figure 3, A and B).

### Hyal-2 OEx in trophoblast cells resulted in impaired uterine NK cells recruitment and function.

**Figure 7:**
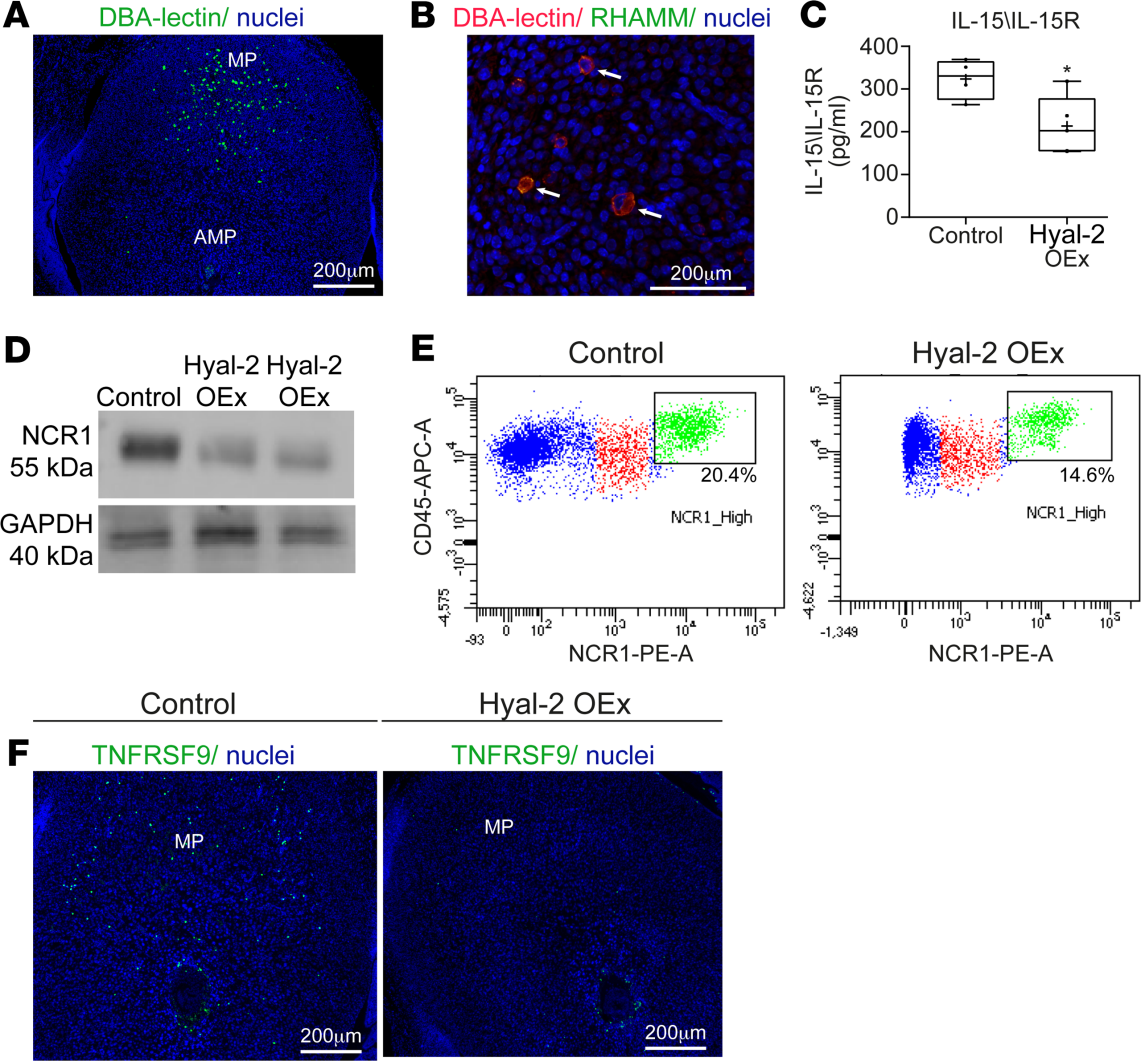
Trophoblast Hyal-2 overexpression impairs uterine NK cell recruitment and function. (**A**) Representative images of DBA^+^ uterine NK cells staining in E6.5 decidua (*n* = 4 dams in each group). (**B**) Immunofluorescence of colocalized DBA^+^ uterine NK cells with hyaluronan receptor RHAMM at E6.5 decidua. (**C**) Impaired uterine NK cell recruitment demonstrated by decreased IL-15/IL-15R complexes detected by ELISA (*n* = 5 dams in each group 323.4 ± 23.49 pg/mL [control]; 213.65 ± 30.43 pg/mL [overexpression], *P* = 0.2). (**D**) Western blot analysis for NCR-1 in E6.5 decidual extracts demonstrated decreased accumulation of NCR-1–expressing NK cells in Hyal-2 OEx foster dams (*n* = 3 dams, 2 implantation sites in each group). (**E**) Flow cytometry analysis of E6.5 implantation sites harvested from foster dams of both groups. Note decreased ratio of CD45^+^NCR-1^+^ population in the Hyal-2 OEx group. (**F**) Immunofluorescence of TNFRSF9 expressed by uterine NK cells, in the mesometrial pole in E6.5 decidua. The statistical analysis applied was Student’s *t* test (**C**).

Differentiated Dolichos biflorus agglutinin (DBA) reactive uterine NK cells were detected in the mesometrial pole of E6.5 decidua (Figure 7A). The latter demonstrated their potential of binding hyaluronan by expressing RHAMM (Figure 7B). Alongside the altered decidual vasculature observed in foster dams carrying Hyal-2 OEx embryos, uterine NK cell recruitment was largely impaired, as indicated by the reduction in the IL-15–IL-15R binding assay (Figure 7C). This effect, combined with the decrease in proliferating DBA^+^ uterine NK cells, evidently brought about a decrease in the abundance of uterine NK cells (Figure 7, D and E, and Supplemental Figure 3C). Nevertheless, the levels of IFN-γ, a major regulatory cytokine produced by uterine NK cells (14), was not decreased in decidua of Hyal-2 OEx embryos (Supplemental Figure 3D). The diminished accumulation of uterine NK cells in the mesometrial pole, and the decreased expression of uterine NK cell classification marker TNFRSF-9 (Figure 7F), correlated with the impaired VSF enlargement observed above (Figure 6I), thereby pointing out that the diminished decidual hyaluronan inflicts the circumferential damage upon differentiated DBA^+^ uterine NK cells and their vascular remodeling potency.

### Augmented hyaluronan degradation severely alters uterine NK cell differentiation and homeostatic shift.

**Figure 8:**
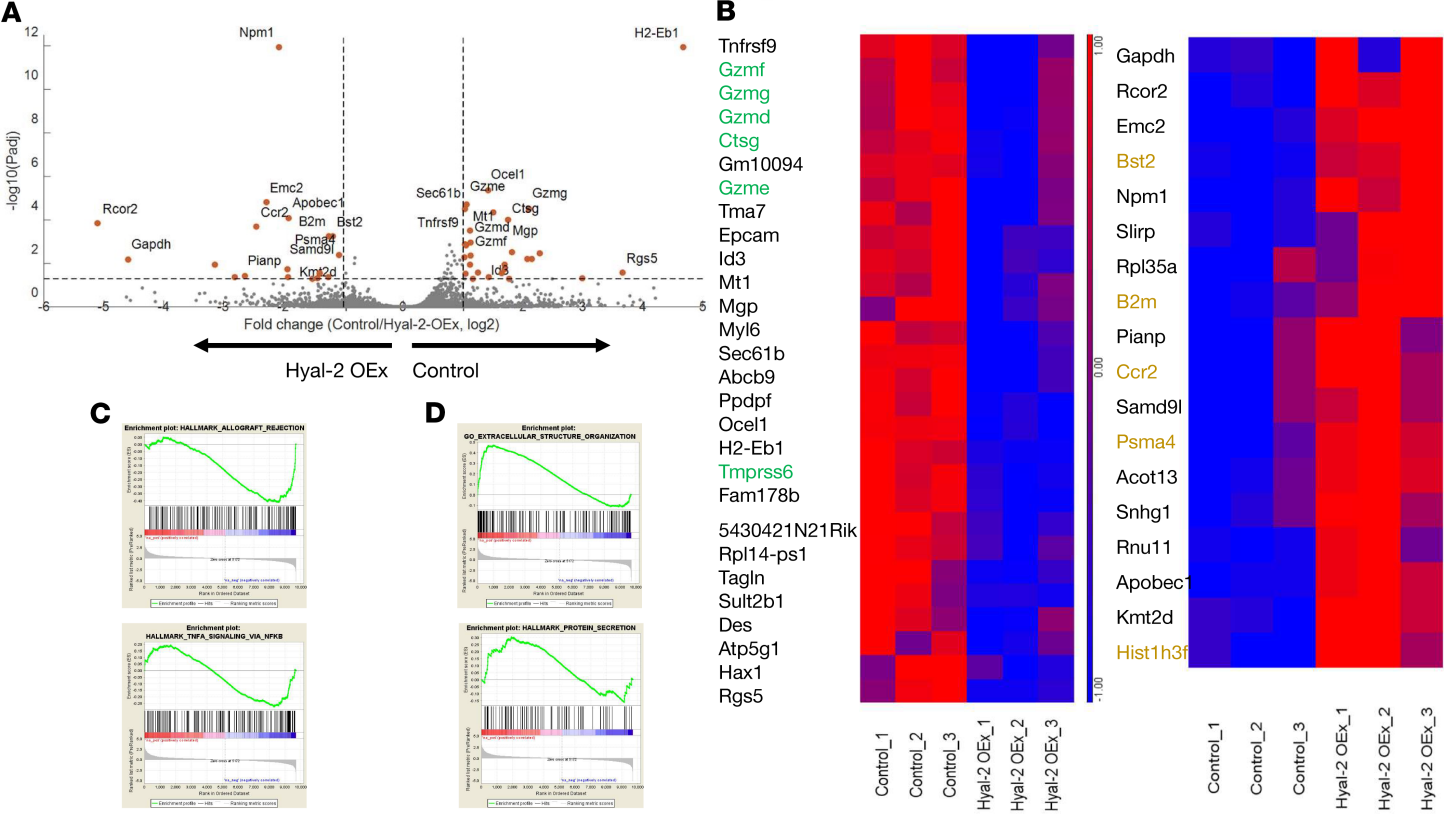
Uterine NK cells transcriptome is dysregulated in foster dams carrying Hyal-2 OEx embryos. NCR1^+^ uterine NK cells were sorted from E6.5 decidua of surrogate mice (*n* = 3 dams; pools of 4 implantation sites each). (**A**) Volcano plot of all genes detected in RNA sequencing analysis. All points above the gray dotted horizontal line are statistically significant. Raw *P* values were adjusted for multiple testing using the procedure of Benjamini and Hochberg. Genes associated with antiviral and innate immune response are indicated in Hyal-2 OEx. Genes associated with uterine NK cells classification are indicated in Control. (**B**) Heatmap of differentially expressed (DE) genes in uterine NK cells sorted from control dams. Bolt DE genes in green were associated serine-type peptidase activity, characteristic of uterine NK cells by GO: molecular function. Bolt DE genes in orange were associated Cytokine Signaling in Immune System by GO: Pathways. (**C**) GSEA analysis of uterine NK cells, sorted from Hyal-2 OEx dams, tested for transcriptional enrichment in the above-indicated pathways. Genes clustered above the dotted line are significantly enriched. (**D**) GSEA analysis of uterine NK cells, sorted from control dams, tested for transcriptional enrichment in the above-indicated pathways. Genes clustered above the dotted line are significantly enriched.

Transcriptome profiling of NCR-1–expressing uterine NK cells sorted from E6.5 decidua revealed 46 differentially expressed genes between uterine NK cells, sorted from dams carrying control embryos and those sorted from foster dams carrying Hyal-2 OEx embryos (Gene Expression Omnibus [GEO] accession no. GSE156979; https://www.ncbi.nlm.nih.gov/geo/query/acc.cgi?acc=GSM4749279). Expression of genes associated with the innate immune response was increased in uterine NK cells from mice carrying Hyal-2 OEx embryos, as opposed to genes associated with unique uterine NK cell classification, differentiation, machinery of protein translation and secretion, and immunomodulation, whose expression was strongly decreased (Figure 8A). Functional analysis of sorted uterine NK cells from dams carrying Hyal-2 OEx embryos revealed significantly reduced protein translation machinery, intra- and extracellular secretion machinery, vascular remodeling factors, and granzyme-mediated apoptotic signaling, traditionally associated with uterine NK cells (Ctsg, Gzmc, Gzme, Gzmf, Gzmg, Sec61b, Spp1, Tnfrs9) (Figure 8, B and C). Functionality of uterine NK cells sorted from those dams differed significantly, revealing enrichment of multiple genes, which regulate the inflammatory response and take part in allograft rejection signaling (Tnf, Ccr2, Samd9l, B2m, Npm1, Ly86), diverting from their classification as immunomodulatory NK cells (Figure 8D).

### HAS-2 OEx in trophoblast cells resulted in decreased permeability of decidual blood vessels, leading to early embryonic lethality.

**Figure 9:**
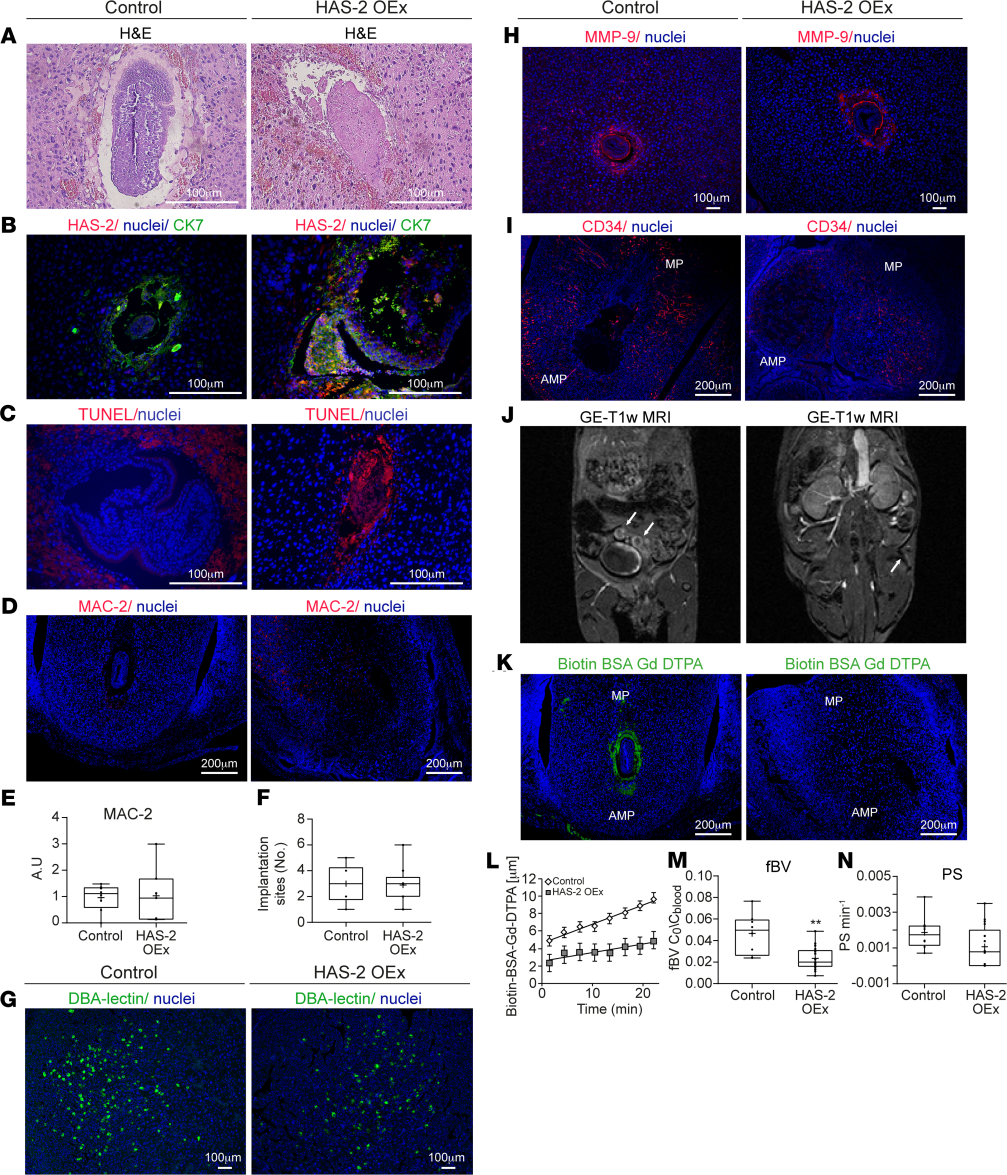
Trophectoderm overexpression of HAS-2 resulted in early embryonic lethality and attenuated decidual angiogenesis. (**A**) H&E staining of implantation sites revealed embryo resorption upon HAS-2 OEx (*n* = 4 dams). (**B**) Remnants of embryo overexpressing HAS-2 by trophoblast cells (CK-7) as opposed to decidual cells expressing HAS-2 in the embryonic niche in the control (*n* = 3 dams). (**C**) Profound embryonic cell death indicated by TUNEL staining was detected upon HAS-2 OEx, (*n* = 3 dams). (**D**) Similar pattern of MAC-2^+^ macrophages, confined to the mesometrial pole, away from the embryonic niche, observed in both groups (*n* = 3 dams). (**E**) Quantification MAC-2 staining by fluorescent microscopy (1.066-fold change ± 0.18 [control]; 0.41 [overexpression]; *n* = 5 dams, 7 implantation sites, *P* = 0.879). (**F**) Number of embryo implantation site per dam was assessed, at E6.5 by gross morphology inspection, as well as by examination of histological sections (3 ± 0.63 [control]; 2.88 ± 0.61 [overexpression], *P* = 0.88) (*n* = 6 dams in control; 8 dams in HAS-2 OEx). (**G**) Representative image of DBA^+^ NK cells in E6.5 decidua (*n* = 3 dams). (**H**) Representative image of MMP-9 in E6.5 decidua (*n* = 3 dams). (**I**) Impaired development of CD34^+^ newly formed blood vessels was observed in pregnant mice carrying HAS-2 OEx embryos, reflected by confinement of vessels away from the embryonic niche (*n* = 3 dams). (**J**) T1 weighted GE-MRI of embryo implantation sites, acquired from pregnant mice at E6.5 30 minutes after administration of biotin-BSA-GdDTPA. Little accumulation of biotin-BSA-GdDTPA was observed in the in dams carrying HAS-2 OEx embryos in comparison with control (white arrows indicate implantation sites; *n* = 6 dams). (**K**) Visualization of hyperpermeable blood vessels in the embryonic niche was achieved by staining of biotin-BSA-GdDTPA, 40 minutes after i.v. injection. Hyperpermeable vessels were not detected at HAS-2 OEx decidua in contrast to those detected in the control group (*n* = 3 dams). (**L–N**) These observations were consistent with DCE-MRI of biotin-BSA-GdDTPA (**L**), fBV (**M**) (0.041 ± 0.005; 0.023 ± 0.002; *P* = 0.004) and PS (**N**) (0.0017 ± 0.0003; 0.0009 ± 0.00031; *P* = 0.2) (control: 5 dams 9 implantation sites; HAS-2 OEx: 6 dams 19 implantation sites). The statistical analysis applied was Student’s *t* test (**E**, **F**, **M**, and **N**).

To further confirm the contribution of hyaluronan metabolism to the success of implantation, lentiviral blastocyst infection was also employed for HAS-2 overexpression (HAS-2 OEx). As expected, the deposition of periembryo hyaluronan was enhanced upon the transfer of these blastocysts to pseudopregnant mice (Figure 4). Nevertheless, while no effect on either infiltration of inflammatory macrophages or on the number of implantation sites (Figure 9, D–F) was observed, these pregnancies resulted in multiple embryo resorptions (Figure 9, A–C, and Supplemental Figure 2G). Unlike Hyal-2 OEx decidua, amplified production of hyaluronan by the trophoblast did not affect the abundance of DBA^+^ uterine NK cells and the expression of the ECM modifier MMP-9 (Figure 9, G and H). Furthermore, upon the transfer of HAS-2 OEx blastocysts, implantation sites showed impaired formation of new blood vessels (Figure 9I) and decreased accumulation of i.v. injected biotin-BSA-GdDTPA, as detected by MRI, 20 minutes after its administration (Figure 9J). This decrease was further validated by histological staining of the contrast agent at 40 minutes after injection (Figure 9K). Moreover, dynamic contrast–enhanced (DCE) MRI analysis of pregnant mice carrying HAS-2 OEx embryos revealed vascular changes, which were opposite to those induced by Hyal-2 OEx (Figure 9L), with significantly decreased fractional blood volume (fBV) (Figure 9M) and blood-vessel permeability (Figure 9N).

## Discussion

During the postimplantation stage, before the formation of a functional hemochorial placenta, decidual vasculature provides the embryo with oxygen and essential nutrients by diffusion. Analyses of the embryo-maternal interface demonstrated the confinement of decidual angiogenesis to the outskirts of the implantation site in the antimesometrial pole and its absence in the mesometrial pole (5). Interestingly, this compartmentalization takes place despite the flux of paracrine, hypoxia-induced, proangiogenic signals secreted from uterine decidual cells adjacent to the embryo, facing both decidual poles (5). Furthermore, the maternal primary decidual zone, at the immediate vicinity of the embryo, is characterized by impermeable vasculature. The goal of the present work was to decipher the mechanism responsible for the maintenance of a vascular permeability barrier at the feto-maternal interface of the postimplantation embryo during the early stages of pregnancy and to further unveil its significance to pregnancy outcome.

Our experiments show that dynamic deposition and degradation of hyaluronan throughout embryo implantation correlated with decidual angiogenesis and vascular remodeling. Lineage-specific and reciprocal genetic manipulations of trophoblast expression of key hyaluronan metabolic enzymes provided evidence for the discrete spatiotemporal roles for hyaluronan, as well as its metabolites, which are indispensable for proper development of the embryo. Specifically, our findings support the role of high–molecular weight hyaluronan as a negative angiogenic morphogen during early pregnancy, further suggesting that hyaluronan breakdown products, generated upon its degradation by hyaluronidases, promote vascular permeability via the VEGF/VEGFR-2 signaling pathway, to support perfusion to the developing embryo. The temporal fine-tuning of conversion of the full-length hyaluronan into its degradation products is governed by progesterone signaling. The effect of hyaluronan in vascular remodeling and decidual homeostasis involves uterine NK cells, and its contribution to trophoblast invasion is assisted by MMP-9.

To assess the involvement of progesterone in the frame of physiological decidual hyaluronan metabolism, we pharmacologically inhibited the downstream actions of its receptor, after attachment is established, in order to allow the onset of gestation. This treatment lowered the expression of both Hyal-1 and Hyal-2 at E5.5, together with that of VEGF-A, previously shown as a target of decidual PR and a regulator of the postattachment angiogenic reaction in mice (5). Thus, in addition to its effect on decidual angiogenesis, PR signaling positively regulated hyaluronidase expression in primary decidual cells, enabling invasion of trophoblast cells to the embedding maternal stroma. The latter coincides with previous reports of increased hyaluronan cleavage and upregulation of Hyal-2 in cycling ovine endometrium (38).

Expression of both hyaluronan-synthesizing enzymes, as well as hyaluronan’s degrading enzymes, in the feto-maternal interface was prominent throughout the postimplantation period. Specifically, Hyal-2 and HAS-2 were observed in the giant as well as the cytotrophoblast cells in the ectoplacental cone at E6.5. Subsequent to its function in hyaluronan clearance, Hyal-2 — which was also observed in primary uterine decidual cells, adjacent to the embryonic niche — generates intermediate hyaluronan fragments of approximately 20 kDa, which are proangiogenic (25). Furthermore, 3 receptors for hyaluronan were distributed in the vicinity of the implanted embryo at E6.5, either at mesometrial orientation or throughout the primary decidual zone of RHAMM, LYVE-1, and CD44. To study the impact of periembryo hyaluronan metabolism, we used lentiviral infection generating either Hyal-2 OEx or HAS-2 OEx blastocysts, targeted exclusively to their trophectoderm cells. Enhanced degradation of hyaluronan at the decidual-trophoblast interface, by Hyal-2 overexpression, resulted in smaller decidua, cell death in the embryonic niche, and abnormal morphology of the embryos.

Similar to connective tissue homeostasis and cancer metastasis (33, 40), Hyal-2 OEx by trophoblast cells induced both zymogen as well the mature form of MMP-9 in a similar pattern to that previously demonstrated in vitro (33). This response apparently resulted in excess of lateral and mesometrial stromal invasion at E6.5, accompanied by impaired uterine NK cell recruitment. This genetic manipulation was also accompanied by prominent ectopic angiogenesis proximal to the ectoplacental cone, manifested by local elevation in fBV and blood-vessel permeability, as well as leakage of maternal blood into the embryonic niche. The prominent increase in blood volume can be attributed to the distinctive proangiogenic effects of Hyal-2–mediated hyaluronan degradation products. The latter could potentially act through either the release of ECM-bound VEGF secondary to the upregulated MMP-9 activity, as previously demonstrated in carcinogenesis (37), or directly via upregulated expression of VEGF and its receptor VEGFR-2, observed herein and also demonstrated during other distinct vascular remodeling events (29, 30, 41). Furthermore, Hyal-2 OEx resulted in compromised recruitment and function of uterine NK cells, responsible for VSF formation, via enlargement and maturation of mesometrial vasculature and prevention of vessel sprouting, as previously demonstrated during postimplantation development in mice (5, 17, 42). Additionally, VEGFR-3 — another regulator of VSF formation (5) — has been demonstrated as an attenuator of VEGF/VEGFR-2–induced vascular permeability via inhibition of VEGFR-2 (9). Therefore, we cannot rule out the contribution of decreased VEGFR-3 to the mesometrial induction of VEGFR-2, resulting in vascular hyperpermeability.

The second trimester of human pregnancy is characterized by prominent invasion of differentiated extravillous trophoblast (EVT) cells into the maternal decidua. Invasive EVT cells possess a key role in these substantial tissue-transforming events, including ECM and vascular remodeling, and this enables the formation of nonvasoactive vessels that permit efficient transport of maternal blood to the intervillous space to provide oxygen and nutrients to the developing embryo (43). Interestingly, augmented trophoblastic hyaluronidase expression resulted in impaired expansion of mesometrial vascular spaces, notably responsible for constant transport to the mouse embryo. The latter may imply that hyaluronan is a potential mediator of spiral arteries remodeling during early human gestation.

Transcriptome and functional analysis of uterine NK cells, sorted from foster dams, indicated a prominent shift in uterine NK cell programming in surrogate mice carrying Hyal-2 OEx embryos, alongside impaired recruitment and vascular remodeling attributes. The effect was most prominent in DBA^+^ uterine NK cells, demonstrated as “exutero” in origin, as opposed to the DBA^–^ uterine NK cells, which comprise the preexisting NK cell population before implantation at E4.5 (44). The decrease in IL-15–IL-15R binding and the unchanged IFN-γ levels, alongside decreased proliferation of DBA^+^ uterine NK cells, may reflect the differential transformation in the gestational NK cell milieu (14, 45). The latter could be of great clinical significance due to the documented role of IL-15 in the maturation of uterine NK cells following endometrial scratching during fertility treatments (46). Furthermore, general NCR-1^+^ uterine NK cells displayed a phenotype similar to that of peripheral NK cells, depicted by decreased expression of vascular remodeling factors, immunomodulatory molecules, and classification markers, which are usually upregulated in uterine NK cell preplacentation (e.g., Rgs5, Tnfrsf9, Ocel1, Gzmg, Ido3) (16, 47, 48). Interestingly, uterine NK cells sorted from mice carrying Hyal-2 OEx embryos acquired an antiviral-associated phenotype, exhibiting a local enhanced innate immune response. The latter might be an intermediate phenotype as a result of impaired differentiation or an active response to excess trophoblast invasion, observed in Hyal-2 OEx decidua. Hyaluronan receptors, expressed by differentiated uterine NK cells in the mesometrial pole, may serve as sensors for homeostatic perturbation during decidual homeostasis following implantation.

Due to the classical role of hyaluronan as a biological glue (20), it has been hypothesized that it facilitates attachment of the preimplantation embryo. This claim was supported by enhanced attachment of isolated, preimplantation blastocysts to hyaluronan-rich matrix (49) and further challenged by the use of hyaluronan-enriched blastocyst transfer medium with the aim of improving blastocyst adherence, which revealed conflicting outcomes (50, 51). Interestingly, Hyal-2 OEx enhanced implantation rate. This observation may reflect the potency of low–molecular weight hyaluronan to induce cellular motility and invasion, as previously described for tumor cells, fibroblasts, and endothelial cells (34, 52–55). Along this line, RHAMM — a receptor for low–molecular weight hyaluronan, which is an efficient mediator of tumor cell invasion (56) — was prominently expressed at the attachment interface. Moreover, the local induction of MMP-9 expression in the trophectoderm, as a result of Hyal-2 overexpression, may have contributed to the increased implantation rate. It has been reported that low–molecular weight hyaluronan–stimulated expression of proinflammatory cytokines and chemokines triggers sterile inflammation and activates murine proinflammatory M1 macrophages (23, 57). Indeed, Hyal-2 OEx resulted in increased accumulation of CD11b^+^F4/80^+^-activated macrophages in the entire decidua and, particularly, in the embryonic niche.

Augmentation of hyaluronan was induced by overexpression of HAS-2 in embryonic trophoblast cells. HAS-2 OEx did not modify the number of embryo implantation sites, but it resulted in profound embryonic cell death at E6.5. Importantly, while overexpression of either HAS-2 or Hyal-2 brought about implantation failure, each of these treatments led to opposing vascular phenotypes. HAS-2 OEx resulted in decreased angiogenesis and reduced blood volume fraction, and it attenuated permeability of maternal vessels, thus increasing the avascular niche of the implantation site and enlarging the maternal-embryo barrier. Despite the typical decidual morphology, few intact embryonic structures were observed. These results underscore the importance of a fine-tuned maternal-embryo barrier mediated by hyaluronan deposition and degradation so as to maintain embryo integrity, and they assure accurate diffusion distance and spatial organization of trophoblasts, endothelial cells, and immune cells.

Our study shows that hyaluronan acts as an ECM-based dynamic mediator of the primary maternal-embryo barrier for the developing embryo before placentation and is crucial for decidual morphogenesis. Furthermore, our findings highlight a critical role for hyaluronan as a vascular morphogen, which acts via recruitment and function of uterine NK cells, in the frame of gestational vascular adaptations. These findings implicate hyaluronan metabolism as a key regulator for the successful establishment of pregnancy.

## Methods

### Animals.

C57BL/6J female mice (6–12 weeks old; Envigo) were mated with Myr-Venus homozygote males (58). These hemizygote Myr-Venus embryos were used for histological analysis of hyaluronan metabolism. ICR males and females (8–12 weeks old; Envigo) were mated and assessed for the occurrence of vaginal plugs on the following day. These mice were used for gene expression analysis during early pregnancy, as well as for MRI experiments.

### IHC.

For information on IHC, see Supplemental Methods.

### Gene expression analysis.

RNA extraction was performed according to the manufacturer’s protocol (PerfectPure RNA tissue kit, 5 Prime) for all analyses. Then, cDNA production followed the manufacturer’s protocol with the High Capacity cDNA Reverse Transcription kit (Applied Biosystems). Real-time PCR was performed to test the different HAS and hyaluronidases isozymes and the expression of 2 of the hyalhedrins (TSG-6 and Versican) in embryo implantation sites during early pregnancy (E3.5–E6.5). Mouse B2M was used as a reference gene in all experiments. The primers used are listed in Supplemental Table 1. Real-time PCR was performed with SYBR Green (Roche) in a Light cycler 480 machine (Roche), according to manufacturer’s protocol.

### In vivo DCE MRI of embryo implantation sites.

MRI experiments were performed at 9.4 T on a horizontal-bore Biospec spectrometer (Bruker) using a linear coil for excitation and detection (Bruker). The animals were anesthetized with isoflurane (3% for induction, 1%–2% for maintenance; Abbott Laboratories) in 1 L/min oxygen, delivered through a muzzle mask. Respiration was monitored, and body temperature was maintained using a heated bed. The pregnant mice were serially scanned at E6.5 after transgenic embryo transplantation. Three-dimensional gradient echo (3D-GE) images of the implantation sites were acquired before, and sequentially, for 40 minutes after i.v. administration of the contrast agent. A series of variable flip angle, precontrast T_1_-weighted 3D-GE images were acquired to determine the precontrast R_1_ (repetition time [TR]: 10 msec; echo time [TE]: 2.8 msec; flip angles 5°, 15°, 30°, 50°, 70°; 2 averages; matrix, 256 × 256 × 64; field of view [FOV], 35 × 35 × 35 mm^3^). Postcontrast images were obtained with a single flip angle (15°). During MRI experiments, the macromolecular contrast agent biotin-BSA-GdDTPA (80 kDa; Symo-Chem), 10 mg/mouse in 0.2 mL of PBS, was injected i.v. through a preplaced silicone catheter inserted into the tail vein.

The MRI scans allowed quantification of the fBV and the permeability surface area product (PS) of embryo implantation sites, as previously reported (6). In brief, the change in the concentration of the administered biotin-BSA-GdDTPA over time (*Ct*), in the region of interest, was divided by its concentration in blood (*Cblood*; calculated in the region of interest depicting the vena cava, also acquired during MRI, and extrapolated to time 0). Linear regression of these temporal changes in *Ct*/*Cblood* yielded 2 parameters that characterize vascular development and function: (a) fBV (fBV = *C0*/*Cblood*), which describes blood-vessel density and is derived from the extrapolated concentration of the contrast agent implantation sites, at time zero, divided by the measured concentration in the vena cava, approximately 5 minutes after i.v administration*,* and (b) *PS* = ([*Ct – C0*]/[*Cblood* × *t*]), which represents the rate of contrast agent extravasation from blood vessels and its accumulation in the interstitial space and which is derived from the slope of the linear regression of the first 15 minutes after contrast agent administration (*t* = 15). Mean fBV and PS were calculated separately for single implantation sites, considering homogeneity of variances between mice.

At the end of the MRI session, embryo implantation sites were harvested and immediately placed in 4% PFA after sacrificing the pregnant mice by cervical dislocation.

### PR pharmacological blockade.

Pregnant mice (late E4.5) were administered i.p. with RU486 (8 mg/kg, MilliporeSigma) and sacrificed at E5.5. Pregnant uteri were either examined via histological analyses or lysed and subjected to Western blot.

### Generation of transgenic trophoblasts in murine blastocysts.

Lentiviral vectors were constructed to produce lentiviruses expressing mouse Hyal-2, and mouse HAS-2. Mouse *Hyal-2* (GeneBank accession no. NM_010489.2; https://www.ncbi.nlm.nih.gov/nuccore/NM_010489.2) was isolated from uterine cDNA by PCR using a sense primer carrying a human influenza hemagglutinin (HA) tag and restriction sites for AgeI and the antisense primer for SfiI (Supplemental Table 2). Mouse *HAS-2* (GeneBank accession no. NM_008216.3; https://www.ncbi.nlm.nih.gov/nuccore/NM_008216.3) was isolated from uterine cDNA by restriction-free cloning, using primers containing complementary overhangs to the designated target vector (Supplemental Table 2). The purified PCR products were cloned into the lentiviral expression vector pCSC-SP-PW-IRES/GFP (provided by Alon Chen, Department of Neurobiology, The Weizmann Institute of Science, Rehovot, Israel). Recombinant lentiviruses were produced by transient transfection in HEK293FT cells (Invitrogen), as described earlier (59), using 3 envelope and packaging plasmids and one of 3 viral constructs: (a) pCSC-SP-PW-Hyal-2-IRES/EGFP (Hyal-2 OEx), (b) pCSC-SP-PW-HAS-2-IRES/EGFP (HAS-2 OEx), or (c) pCSC-SP-PW-IRES/EGFP (control). Briefly, infectious lentiviruses were harvested at 48 and 72 hours after transfection, filtered through 0.45 mm–pore cellulose acetate filters, and concentrated by ultracentrifugation at 39,200*g*, 4°C for 2.5 hours. Lentiviral supernatant titers were determined by Lenti-X p24 Rapid Titer Kit (Supplemental Table 3) according to manufacturer’s protocol (Takara Bio USA).

### Validation of gene overexpression.

ES-2 cells — an ovarian clear cell carcinoma cell line — were used for validation of both Hyal-2 OEx and HAS-2 OEx after infection with lentiviral vectors. Validation was conducted using Western blot analysis (with the antibodies used for IHC; Supplemental Methods). Blastocysts from all 3 groups were stained by whole-mount incubation with antibodies against Hyal-2 and HAS-2, stained with species-specific secondary antibodies, counterstained with Hoechst (Invitrogen), subsequently mounted in mineral oil, imaged using spinning disk 386 confocal microscope and 710 confocal microscope (Zeiss, Cell Observer SD), and quantified by ImageJ software (NIH).

### Mice and lentiviral transduction.

WT ICR females were superovulated by s.c. injection of pregnant mare’s serum gonadotropin (PMSG) (MilliporeSigma) (5 units) followed 48 hours later by i.p. injection of human chorionic gonadotropin (hCG) (MilliporeSigma) (5 units) and then mated with WT ICR males. Morulae-stage embryos were collected from the females at E2.5 and then incubated in KSOM medium (produced in-house) to obtain expanded blastocysts. Zona pellucida was removed in acidic Tyrode’s solution (60). Next, 15–30 embryos were incubated with lentiviruses, described above, in KSOM for 5 hours. The transduced blastocysts were washed 4 times and then implanted into pseudopregnant ICR females generated after mating with vasectomized ICR males. We transplanted about 10 blastocysts into each mouse using Non-Surgical Embryo Transfer kit (NSET) (ParaTechs). Imaging of dissected E6.5 infected embryos was performed using a fluorescent Nikon ECLIPSE Ti2 microscope.

### Flow cytometry analysis.

Decidual leukocytes were isolated from decidua harvested from foster dams at E6.5, subjected to mechanical fragmentation, and followed by enzymatic digestion as previously described (61). Briefly, tissues were suspended in 1 mL accutase (MilliporeSigma, A6964) and incubated for 15 minutes at 37°C. Next, cells were sequentially filtered through a 100 μm cell strainer, washed with ice cold sorting buffer (PBS supplemented with 0.2 mM EDTA [pH8] and 0.5% BSA), centrifuged (5 minutes, 4°C, 300*g*), and resuspended in ice cold sorting buffer. Then, cells were stained with PE-coupled antibody to mouse NCR1 (29A1.4, BioLegend), APC-coupled antibody to mouse CD45 (30-F11, BioLegend), or CD45 (103115, APC/Cy7, BioLegend), CD11b (12-0112-83, PE, eBioscience), CD11c (48-0114-82, EF450, eBioscience), F4/80 (17-4801-82, APC, eBioscience), and Ly6c (128012, Percp/Cy5.5, BioLegend). Cells were then passed through a 70 μm mesh and washed in FACS buffer (2Mm EDTA, 0.5% BSA in PBS). Cells were then subjected to flow cytometry by a SORP-FACSAriaII machine using a 70 μm nozzle, and they were analyzed using BD FACSDIVA (BD Biosciences) and FlowJo.

### Transcriptomics sequencing.

Massively Parallel Single-Cell RNA-seq library preparation (MARS-seq) NK cell Libraries were prepared as previously described (62). In brief, 20,000 NCR1^+^ cells were sorted into 40 μL of lysis/binding buffer, from which mRNA was captured with 12 mL of Dynabeads oligo(dT) (Dynabeads mRNA DIRECT Purification Kit), washed, and eluted at 85°C with 10 mL of 10 mM Tris-Cl (pH 7.5) and processed according to protocol previously developed for single-cell RNA-seq (62). In brief, the samples were barcoded, converted to cDNA, pooled, and linearly amplified by T7 in vitro transcription. mRNA was then fragmented and converted into a sequencing-ready library, which is then tested for quality and concentration (62). MARS-seq libraries were sequenced using an Illumina NextSeq 500 sequencer, at a sequencing depth of ~5 million reads per sample.

### Bioinformatic analysis.

MARS-seq analysis was done using the User-friendly Transcriptome Analysis Pipeline (UTAP) transcriptome analysis pipeline (63) at the bioinformatics unit. Reads were trimmed using cutadapt (https://cutadapt.readthedocs.org/) and mapped to the Mus_musculus genome (UCSC mm10) using STAR (https://github.com/alexdobin/STAR/releases) v2.4.2a with default parameters. The pipeline quantifies the genes annotated in RefSeq (that have expanded with 1000 bases toward 5′ edge and 100 bases toward 3′ bases). Counting was done using htseq-count (http://htseq.readthedocs.io/) (union mode). Genes having minimum 5 UMI-corrected reads in at least 1 sample were considered. Normalization of the counts and differential expression analysis was performed using DESeq2 (http://www.bioconductor.org/packages/release/bioc/html/DESeq2.html) with the parameters: betaPrior=True, cooksCutoff=FALSE, independentFiltering=FALSE. Raw *P* values were adjusted for multiple testing using the procedure of Benjamini and Hochberg.

Functional analysis of differentially expressed genes (FDR < 0.05) was conducted using GeneAnalytics (https://ga.genecards.org) with default settings. The Holm-Bonferroni’s test correction was implemented to detect pathway enrichment and GO: Molecular Functions analyses. Gene Set Enrichment Analysis (GSEA) was performed using GSEA 3.0 with the GSEAPreranked tool (64, 65). Gene names were converted to human gene symbols, and analyzed with default parameters. The Molecular Signature Database (https://www.gsea-msigdb.org/gsea/msigdb), with Biological Processes and Hallmark gene sets, were used to perform pathway enrichment analysis. Gene expression levels (rld) of specific leading edge subsets were visualized as heatmaps using the Partek software.

### ELISA.

Embryo implantation sites were harvested from foster dams at E6.5. ELISA was performed on total extracted protein according to manufacturer instructions for mouse IL-15/IL-15R complex (NBP1-92667, Novus Biologicals) and IFN-γ (MIF00, R&D Systems). Results represent 5 pools of 3 tissues harvested from each foster dam.

### Statistics.

All statistical analyses used in this study were 2 tailed with a similar level of significance (*P* = 0.05) and demonstrated normal values distribution. Beside the 1-way ANOVA followed by post hoc Tukey-Kramer’s test, conducted for gene expression analysis of hyaluronan metabolism during implantation (Supplemental Figure 1) and quantification of absorbed implantation sites (Supplemental Figure 2), all statistical analyses were examined by *t* tests, while assuming homogenous distribution of variances. All statistical analyses were conducted using GraphPad Prism 8.

### Study approval.

All mice used in this study were maintained under specific pathogen–free conditions and handled under protocols approved by the Weizmann IACUC according to international guidelines.

## Author contributions

RH designed research studies, conducted experiments, acquired data, analyzed data, and wrote the manuscript. EG designed research studies and conducted experiments. AC designed research studies, conducted experiments, acquired data and analyzed data. OA conducted experiments and acquired data. SL designed research studies, conducted experiments, and acquired data. OG analyzed data. ME conducted experiments. GC conducted experiments. RE conducted experiments, acquired data, and analyzed data. ND designed research studies and wrote the manuscript. MN designed research studies and wrote the manuscript. BD analyzed data.

## Supplementary Material

supplemental data

## Version 1 11/19/2020

Electronic publication
