# Supplementary material for: Hyaluronan control of the primary vascular barrier during early mouse pregnancy is mediated by uterine NK cells
Source: JCI Insight. 2020 Nov 19;5(22):e135775. doi: 10.1172/jci.insight.135775 (PMC7710306; doi:10.1172/jci.insight.135775)
Supplement: supplemental data [file jciinsight-5-135775-s148.pdf]

Supplemental Information

Hyaluronan Control of the Primary Vascular Barrier during Early Mouse Pregnancy is Mediated by Uterine NK Cells

Ron Hadas[1], Eran Gershon[2], Aviad Cohen[1,3], Ofir Atrakchi[1], Shlomi Lazar[4], Ofra Golani[5], Bareket Dassa[5], Michal Elbaz[2], Gadi Cohen[1], Raya Eilam[6], Nava Dekel[1] and Michal Neeman[1]

Supplementary Methods

Immunohistochemistry. Uterine sections containing embryo implantation sites were fixed in 4% paraformaldehyde (PFA) and embedded in paraffin. For morphological analysis, tissues were stained with hematoxylin and eosin, whereas consecutive sections underwent immunohistochemical staining. The latter included antigen retrieval in citrate (pH=6.0) or EDTA (pH=8.0) buffers in a pressure cooker at 125ºC for 3 minutes. Antigens were washed, blocked with 20% normal horse serum, permeabilized with 0.2% Triton X-100 in PBS, for 1.5 hours at RT, then incubated overnight with the following primary antibodies: goat anti HAS-1 (sc-23145; Santa Cruz Biotechnology**,** Dallas, TX, USA) goat anti HAS-2 (sc-34067; Santa Cruz Biotechnology), rabbit anti Hyal-2 (ab68608; Abcam, Cambridge, UK), rabbit anti Hyal-1 (ab203293; Abcam) goat anti GFP (ab6673; Abcam), rabbit anti CD44 (ab41478; Abcam), rabbit anti LYVE-1 (70R-LR003; Fitzgerald Industries International, Acton, MA, USA), rat anti CD34 (CL8927PE; Cedarlane, Ontario, Canada), Rabbit anti MMP-9 (ab38898; Abcam), Rabbit anti Cytokeratin-8 (NB110-56919; Novus biologicals, CO, USA), Mouse anti Cytokeratin-7 (ab9021;Abcam) and rat anti-mac-2 (CL8942AP; Cedarlane), rat anti mouse VEGFR-3 (14-5988-81; eBioscience, MA, USA),

Rabbit anti VEGFR-2 (CST-9698S, Cell Signaling, MA, USA) and biotin conjugated DBA (L2785; Sigma-Aldrich, Rehovot, Israel), Rabbit anti Ki-67 (275R; Cell Marque, CA, USA). Next, slides were washed and incubated with secondary antibodies, conjugated to biotin against the appropriate species (except for goat anti GFP in the case of double staining, diluted 1:100 in 2% normal horse serum in PBS, for 1.5 hours at RT. Slides were then washed in PBS and incubated in Cy3 or Cy2-conjugated streptAvidin (Jackson Immunoresearch Laboratories, PA, USA), diluted 1:150 in PBS for 45 minutes, at RT. Cells undergoing apoptosis were detected by TUNEL staining (ApopTag; Merck Millipore, MA USA). Slides were counterstained with Hoechst (Invitrogen, Carlsbad, CA, USA) and subsequently mounted. Next, slides were washed and incubated with the appropriate secondary antibodies, diluted 1:100 in 2% normal horse serum in PBS, for 1.5 hours at RT. Then, slides were washed in PBS and incubated in avidin/biotin complex (ABC, Vectastatin, ABC, Vector Labs, CA, USA) in PBS, for 1.5 hours at RT, followed by exposure to DAB (Sigma-Aldrich, Rehovot, Israel). Slides were counterstained with hematoxylin and subsequently mounted. All slides were imaged using a fluorescent Olympus SZX-RFL2 zoom stereo microscope or a Zeiss LSM710 confocal microscope (Zeiss, Oberkochen, Germany).

Ex-vivo separation of glycosaminoglycans and characterization of HA fragments by gel electrophoresis. Uterine glycoseaminoglycans separation was carried out as follows: A pool of 3 implantation sites per time-point, was lyophilized overnight. Wet/dry weight was determined by weighing the samples before and after lyophilization. Samples were digested in 950µl 0.0005% Phenol Red, 100 mM ammonium acetate, ph 7.0 containing 125 mg proteinase K (Roche) for two hours at 60 °C Another 125 mg of proteinase K was added to each tube and incubated for another two hours. The samples were boiled to inactivate the proteinase K and pelleted by centrifugation to remove any undigested material. Samples were subjected to DNA (20U,DNAse1, Roche) and

RNA degradation (192U,RNAse A, Roche), followed by enzyme heat inactivation. Glycoseaminoglycans were precipitated in Ethanol overnight at -20°C, pelleted by centrifugation, re-suspended in 20 µl (Tris/Borate/EDTA [TBE]1x with loading buffer (TBE, 0.02% bromophenol blue and 2M sucrose) to a total of 25 µl. Select-HA LoLadder (Ambion, UK) was used as molecular weight gel standard. Hyaluronan was separated on Tris-glycine Native gels (Any-kD polyacrylamid gel, 8.6^{x}6.7 cm (W^{x}L) (BIO-RAD, Israel)).

Outer and inner buffer chambers were filled with pre-cooled TBE. Gels were run at 300 V for 1 hour, rinsed in water, then transferred to staining solution (0.005% Stains-All (Sigma), 50% ethanol, 50% water) in the dark overnight. Gels were de-stained in water and imaged on a flatbed scanner.

Western blot analysis. Pools of two deciduae per mouse were harvested from Control and Hyal-2 OEx foster Dams. Cells were lysed in RIPA buffer and equal amounts of protein were separated by either 12 % or 8% SDS-PAGE and transferred to nitrocellulose membranes. For detection, we used Rabbit anti MMP-9 (ab38898; Abcam), Rat anti VEGFR-1 (MAB471; R&D, MN, USA), Rabbit anti VEGF (ab52917; Abcam), Rabbit anti VEGFR-2 (55B11; Cell signaling, MA, USA), Rat anti mouse VEGFR-3 (14-5988-81; eBioscience, MA, USA), Goat anti NCR-1 (AF2225; R&D, MN, USA) and Rabbit anti GAPDH (C-2118S; Cell signaling) antibodies.

Quantification of trophoblast invasion. Post-implantation invading trophoblasts were detected by immunolabeling of cytokeratin-8 (CK-8) on paraffin embedded histological section retrieved from Control and Hyal-2 OEx foster Dams. Invading cells were quantified within several rings around the core of the egg-cylinder, from the CK-8 positive visceral endoderm cells, outward, to the decidual stroma. Analysis and final quantification were performed using ImageJ software

(https://imagej.nih.gov/ij/), excluding auto-fluorescent erythrocytes detected by DNA Hoechst labeling.

Clearing and immunolabeling. Surrogate mothers carrying both Hyal-2 OEx and control embryos, were i.v administered with 10 mg/kg ROX (Molecular Probes) conjugated Lycopersicon Esculentum lectin (Vector Laboratories) for detection of functional blood vessels. Ten minutes after administration, mice were euthanized with pentobarbital (CTS Chemical Industries) and were immediately perfused with 1% heparin (heparin sodium Fresenius, Bodene (PTY) limited) in PBS followed by 4% PFA. Embryo implantation sites were harvested, fixed with 4% PFA for 3 days at 4°C, washed three times with PBS, permeabilized with [0.2% Triton X-100(TX-100] for 4h, and placed overnight in blocking solution [10% normal horse serum in PBS containing 0.05% TX-100], incubated in 1:400 dilution of primary rat anti mouse MAC-2 antibody, and 1:200 dilution of rabbit anti GFP antibody (ab6556, Abcam) in antibody cocktail (50% blocking solution/0.05% TX-100/PBSX1) for 1 week at 4°C, washed for 24h with washing buffer (1% blocking solution/0.05% TX-100/PBS, and incubated with a mixture of secondary Cy5 (1:250 dilution, Jackson Immunoresearch Laboratories) in antibody cocktail for another week at 4°C. Following the 24h wash, samples were subjected to a clearing procedure. For tissue clearing, we used a modified protocol based on Chung et al., 2013 and Hama et al., 2011. Briefly, following staining, tissues were removed to hydrogel solution (4% Acrylamide, 0.025% Bisacrylamide, 0.25% Va-044, 4% PFA in PBSX1) for 2 weeks followed by passive clearing process (200mM Boric acid, 4% SDS) for 2 weeks at 37°C. In the next step, samples were placed in ScaleA2 (4M Urea, 10% Glycerol, 0.1% TX-100) for 36h. 3D images and movies were acquired using a Zeiss LSM710 confocal microscope (Zeiss, Oberkochen, Germany).

Supplemental figures

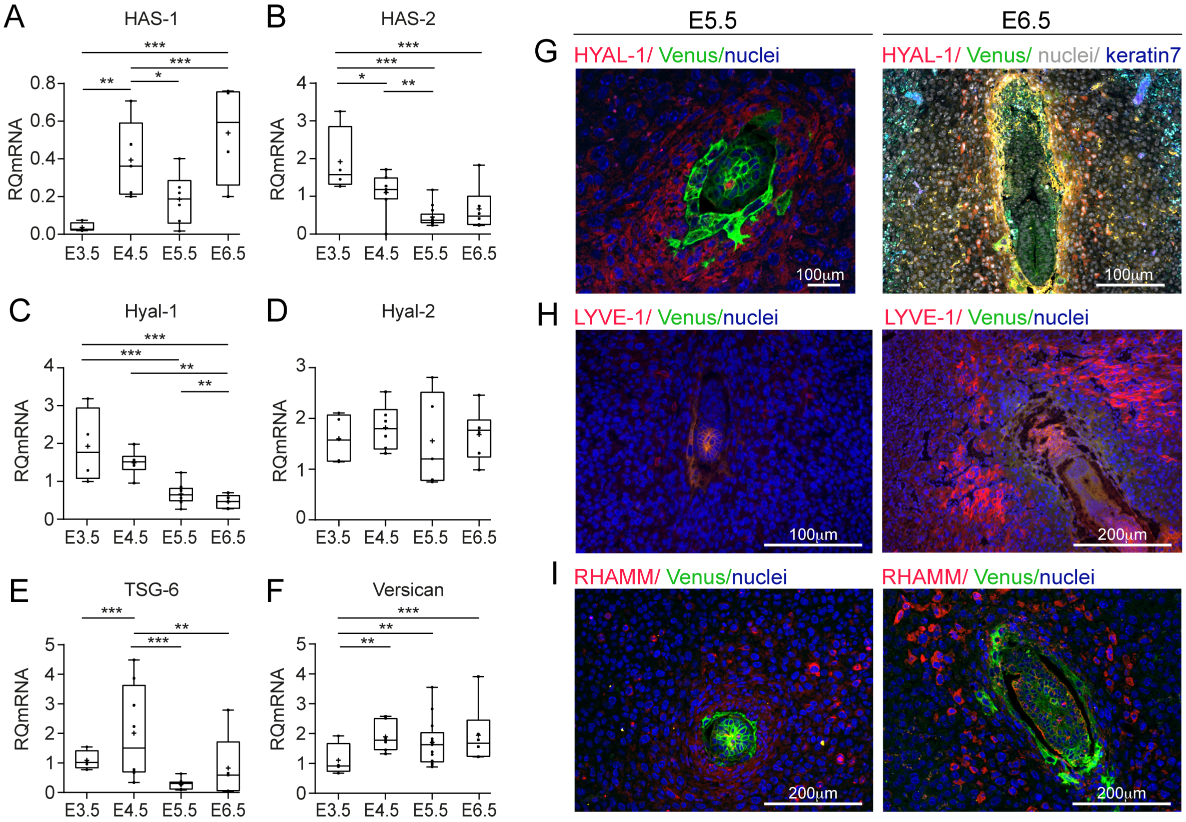

Figure S1. **Hyaluronan metabolism following implantation.** The statistical analysis applied was on-way ANOVA analysis followed by post-hoc Tukey-Kramer's test (A-F). (**A-F**) Equal number of embryo implantation sites were harvested from each pregnant mouse. A total of 4 mice was examined at different time points. RNA was extracted and subjected to real-time PCR analysis. Histological analysis of post-implantation deciduae (E5.5-E6.5) (n=4). (**G**) Distribution of Hyal-1, hyaluronan degrading enzyme, during implantation. (**H**) Representative image of hyaluronan receptor, LYVE-1 decidual distribution, during implantation (n=3 dams). (**I**) Representative images of hyaluronan receptor, RHAMM distribution during implantation in (n=3 dams).

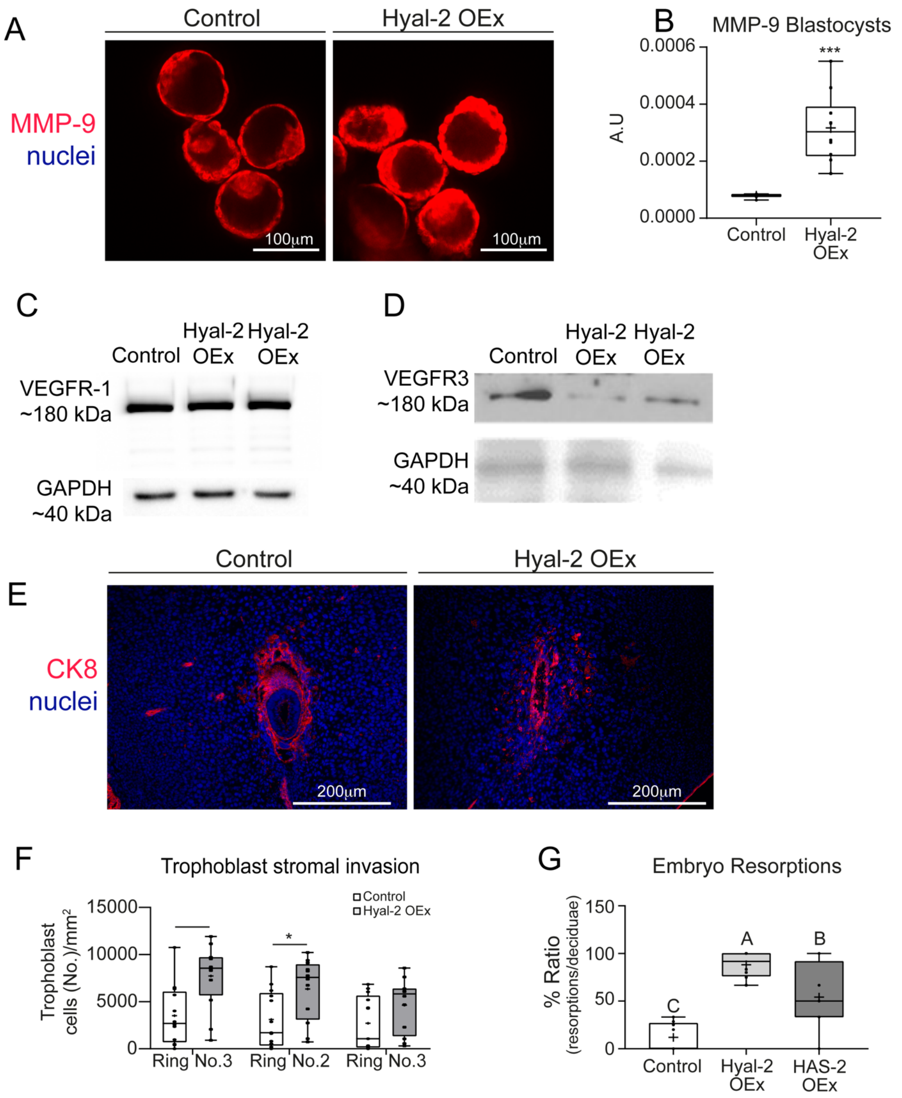

Figure S2. **Hyal-2 over-expression results increased MMP-9 levels and enhanced trophoblast decidual invasion.** The statistical analysis applied were Student's t-test (F) and on-way ANOVA analysis followed by post-hoc Tukey-Kramer's test (G). (**A**) Elevated expression of MMP-9 in blastocysts as a result of Hyal-2 over-expression. (**B**) Quantification of MMP-9 expression following whole mount immunofluorescence of blastocysts (4.04858 fold change±0.03; 0.48 p=0.0001) (n=7 embryos in the control; 10 embryos in Hyal-2 OEx). (**C**) Similar levels of VEGFR-1 in deciduae harvested at E6.5 (n=3 dams 2 implantation sites from each group) (**D**) Elevated expression of VEGFR-3 in deciduae harvested at E6.5 (n=3 dams 2 implantation sites from each group). (**E-F**) Excessive invasion of trophoblast cells detected in Hyal-2 over-expressing trophoblast cells (CK-8) in decidual stroma surrounding the embryo. (**G**) Quantification of embryo

resorption in E6.5 deciduae either by dissection of post implantation embryos or by histological examination (11.87±4.83 for Control; 88.12±4.79 for Hyal-2 OEx; 54.1±4.79 for HAS-2 OEx).

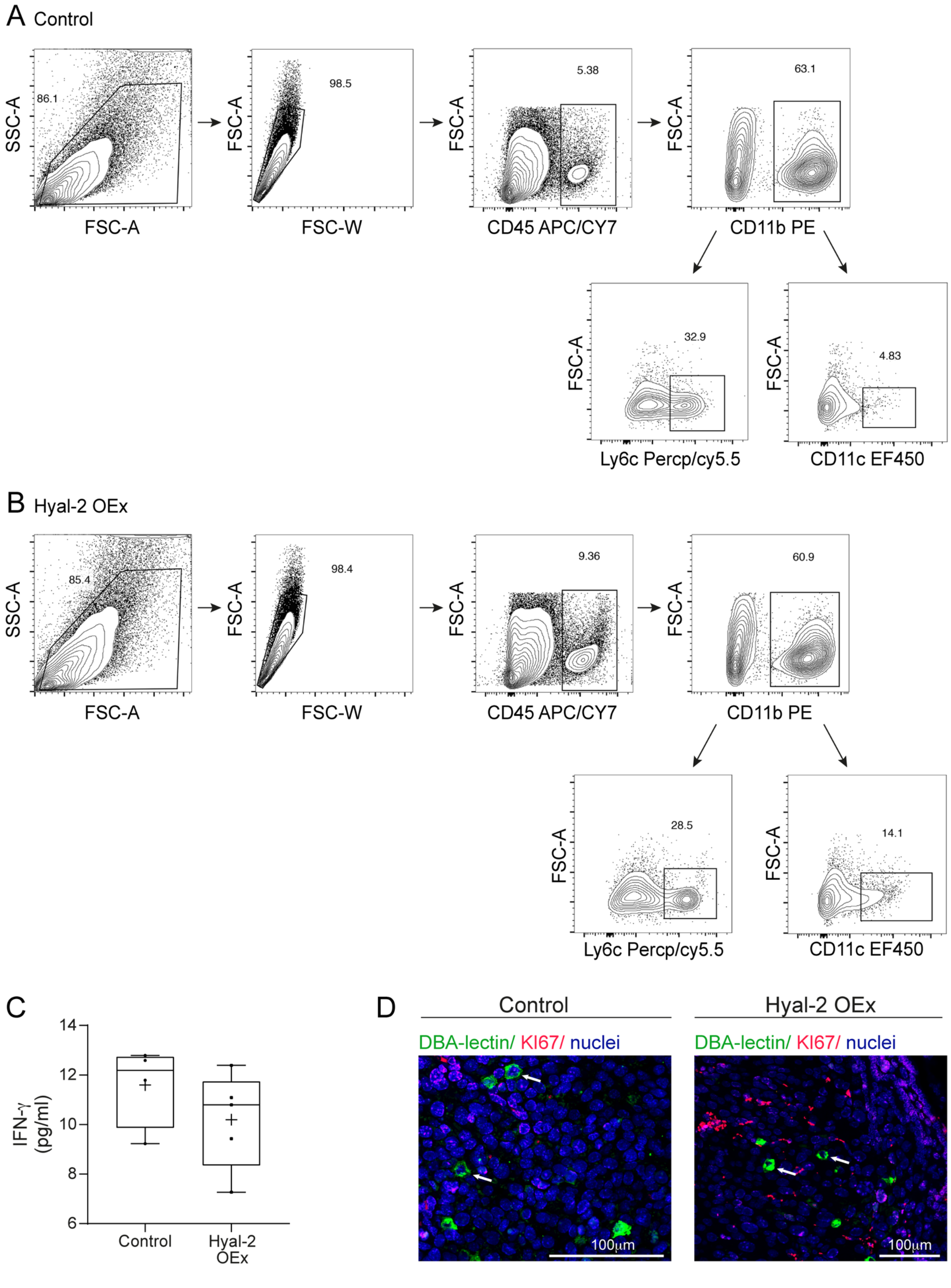

Figure S3. **Augmented hyaluronan degradation transforms the decidual immune milieu following implantation.** (**A-B**) Flow cytometry analysis of CD45+ CD11b+ Ly6C+/CD11c mononuclear

phagocytes cells in E6.5 deciduae (CD45-8.78±0.29; 6.033± 0.12; p=0.004) (n=3 dams in control, n=5 dams in Hyal-2 OEx). (**C**) ELISA detection of IFN-γ (n=4 dams in control 11.59pg/ml±0.86; n=5 dams in Hyal-2 OEx 10.19pg/ml±0.81, p=0.2 (**D**) Immunofluorescence of co-localized DBA+ uterine NK cells with KI67 in E6.5 deciduae.

Table S1. Real Time PCR primers

**Gene**	**Forward primer**	**Reverse primer**
Mouse *HAS*-1	CTACGTGCAGGTCTGTGACTC	GCTCGTTCCACATTGAAGGC
Mouse *HAS-2*	GGCCGGTCGTCTCAAATTCA	ACAATGCATCTTGTTCAGCTCC
Mouse *Hyal*-1	TTCAGTCCTGAGGTTTCCCCA	GGTTGGATACCACGGAACCT
Mouse *Hyal*-2	CTCAGCTGGCGTCATCTTCT	GCCCAGGACACGTTGACTAT
Mouse *TSG-6* (*Tnfaip6*)	AGTGAGCGATGGGATGCCTAT	TCGCTTCGGATCTGTGAAGA
Mouse *Versican* (V-1)	CGTTTTGAGAACCAGACATGC TT	TTGGATGACCACTTACAATCA TATCA

Mouse *B2M*	CCCGCCTCACATTGAAATCC	GCGTATGTATCAGTCTCAGTGG

Table S2. Primers used for cloning

Vector	Insert	Forward primer	Reverse primer
pCSC-SP-PW-IRES/eGFP	HA-*Hyal-2*	ATATACCGGTGCCATGGCTTACCATACGATGTTCCAGATTACATGCGGGCAGGACTAGGTCC	ATATGGCCGCCTGGGCCTCATAAGGTCCAGGTGAGAG
	HAS-2	AGAAGACACCGACTCTAGAGGATCCATGCATTGTGAGAGGTTTCTATGT	GGGGGGGGCGGAATTCTGCAGTCATACATCAAGCACCATGTCA

Table S3. Viral titers measured for lentiviral vectors

Insert	Vector	Viral titer (IFU/ml)
Control	pCSC_SPPWCMV_IRES_GFP	$\geq 1 \times 10^7$
Hyal-2 CDS	pCSC_SPPW_CMV_Hyal-2_IRES_GFP	$\geq 1 \times 10^7$
